# Effects of fireworks on particulate matter concentration in a narrow valley: the case of the Medellín metropolitan area

**DOI:** 10.1007/s10661-019-7838-9

**Published:** 2019-12-03

**Authors:** Carlos D. Hoyos, Laura Herrera-Mejía, Natalia Roldán-Henao, Alejandra Isaza

**Affiliations:** 1 Departamento de Geociencias y Medio Ambiente, Facultad de Minas, Universidad Nacional de Colombia, Sede Medellín, Medellín, Colombia; 2 Sistema de Alerta Temprana de Medellín y el Valle de Aburrá (SIATA), Área Metropolitana del Valle de Aburrá (AMVA), Medellín, Colombia

**Keywords:** Atmospheric boundary layer, Air quality, Atmospheric stability, Black carbon, Fireworks, Particulate matter

## Abstract

The extensive use of fireworks generates large amounts of pollutants, deteriorating air quality and potentially causing adverse health impacts. In Medellín and its metropolitan area, although fireworks are banned during December, their use is widespread during the Christmas season, particularly during the midnight of November 30 (La Alborada) and New Year’s Eve (NYE). It is therefore essential to assess the effects of these celebrations on air quality in the region. Air-quality data from the official monitoring network and a low-cost particulate matter (PM) citizen science project, backscattering intensity (BI) retrievals from a ceilometer network, potential temperature from a microwave radiometer, and information from a radar wind profiler provide an excellent platform to study the spatio-temporal distribution of contaminants resulting from the La Alborada and NYE celebrations. Substantial increases in PM2.5 and PM10 mass concentrations due to La Alborada and NYE, ranging in some cases from 50 to 100 μgm^−3^, are observed in the Aburrá Valley and particularly in the densely populated communes of Medellín, with most concentration changes corresponding to ultrafine and fine particles. The PM increments resulting from fireworks show almost no increase in the net amount of black carbon in the atmosphere. Ceilometer BI profiles show a substantial change immediately after the La Alborada and NYE midnights, confined to the atmospheric boundary layer (ABL). Strong thermal inversions lead to fairly homogeneous increments in BI within the ABL, lasting until the onset of the convective boundary layer. In contrast, weak thermal inversions lead to rapid dispersion of aerosols, allowing them to episodically escape above the ABL.

## Introduction

The massive use of fireworks during short periods generates large amounts of pollutants in the atmosphere, generally within the atmospheric boundary layer (ABL), causing a deterioration in air quality (Lin 2016). The highest recorded hourly concentration of fine particulate matter (PM2.5) corresponds to exposure to firework plumes, nearly reaching 10,000 μgm^−3^ (Joly et al. 2010). Fireworks used in national, regional, religious, and military celebrations and parades, as well as in cultural and sporting events, are composed of oxidant and fuel agents and other components such as agglutinants, coloring agents, smoke, and propellants, all of which, after an oxide-reduction reaction produces different pollutants and particulate matter (PM) into the atmosphere. In addition to the health risks associated with the manipulation of fireworks, the potential for wildfire and infrastructure fire ignition, and the distressing effect on wild and domestic animals because of the loud noise, a high concentration of fireworks-related pollutants during short periods could result in severe health impacts, depending on the PM composition (Bach et al. 1975; Salvi et al. 1999; Licudine et al. 2012; Kloog et al. 2013; Watanabe et al. 2016). The pioneering evidence presented by Bach et al. (1975) indicated that a 170% increase in pollutant concentration was associated with a 113% increase in respiratory illness. One of the potential health risks is associated with perchlorate, common in the vicinity of fireworks-manufacturing sites and firework display sites, contaminating the groundwater and the surface water (Sijimol and Mohan 2014). Perchlorate contamination could disrupt the thyroid and has an impact on ecology (Greer et al. 2002; Sijimol and Mohan 2014). Similarly, toxic byproducts appear as a result of atmospheric reactions between metal oxides and organic fuels (Fleischer et al. 1999).

Several authors have described adverse effects on air quality resulting from the use of fireworks as part of popular festivities around the world. Hourly concentrations of PM2.5 associated with the fireworks of Fourth of July, a national US holiday commemorating the Declaration of Independence, have been reported to be considerably higher than those of the preceding and following days, with an average increase of 21 μgm^−3^ in the 21:00–22:00 local time—LT-period (Seidel and Birnbaum 2015). The Hindu Diwali festival, in India, is characterized by the widespread use of firecrackers constituting a source of primary aerosols, black carbon (BC), organics, and trace gas emission. A two- to threefold increase in pollutants has been measured during these festivities compared with prior days, and coarse particulate matter (PM10) concentration has reached values over 750 μgm^−3^, more than five times higher than during a regular day (Barman et al. 2008; Yerramsetti et al. 2013). In addition to finding a considerable increment in PM10 (1.3- to fourfold increase), Mandal et al. (2012) also found an important increase in the ambient noise. Adverse meteorological conditions during the Diwali festival, characterized by a decrease in the ABL height and the magnitude of surface winds, also play a role in pollutant concentration associated with fireworks (Singh et al. 2010). The pollution generated during this festival has been reported to increase the concentrations of Ba, K, Sr, Mg, Na, S, Al, Cl, Mn, Ca, and EC by factors of 264, 18, 15, 5.8, 5, 4, 3.2, 3, 2.7, 1.6, and 4.3, respectively (Sarkar et al. 2010). Recently, the induced pollution due to Diwali fireworks has been assessed using in situ monitoring sites and MODIS retrievals (Kumar et al. 2016). The results suggest a nationwide increase in PM, over 50%, during the festival days in comparison with their background concentrations.

In China, the world’s largest fireworks producer, their adverse effects on air quality have been reported not only for large urban areas during the Lantern Festival (Wang et al. 2007), with a sixfold increase in PM2.5 reported during 2006, but also for the rural and suburban areas of the Henan and Shandong provinces (Zhang et al. 2017). During Taiwan’s Lantern Festival, PM10 has been recorded to be higher than 150 μgm^−3^, with 60% of the mass corresponding to PM2.5, and the concentration of metallic elements has been up to ten times higher than that of the typical values (Tsai et al. 2012). In Girona, Spain, during the Sant Joan fiesta, there have been reports of increased local background PM2.5 concentrations with Sr, K, Ba, Co, Pb, Cu, Zn, Bi, Mg, Rb, Sb, P, Ga, Mn, As, and Ti increasing 86, 26, 11, 9, 7, 5, 4, 4, 4, 4, 3, 3, 2, 2, 2, and 2 times, respectively (Moreno et al. 2010). During the 2006 soccer World Cup celebrations in Milan, Italy, PM10 concentrations increased by approximately 50%, with a 120-times increase in Sr, 22-times in Mg, 12-times in Ba, 11-times in K, and 6-times in Cu (Vecchi et al. 2008). Dutcher et al. (1999) reported concentrations of fine particles over 150 μgm^−3^ associated with indoor pyrotechnic displays in sporting events, dominated by an increase in K and S, which originate primarily from black powder. In a recent review article, Singh et al. (2019) present compelling evidence of the effect of fireworks on air quality, visibility, and human health, highlighting the fact that pollutant concentrations are usually 2–8 times higher than average during fireworks events, with significant changes in concentrations of elements such as Ba, Cu, Pb, Cr and Sr, and a decrease in visibility by as much as 92%. Singh et al. (2019) also stress the need for more research on the respiratory impacts of fireworks and more evidence from different regions of the world.

In Medellín, Colombia, and its surrounding metropolitan area in the Aburrá Valley, it has become a highly controversial tradition, apparently since 2003, to celebrate the beginning of December and the Christmas season with widespread use of firecrackers and fireworks by a large portion of the population. This tradition has been referred to as “La Alborada,” Spanish for “The Dawn”. The main difference with most of the previously mentioned cases around the globe is that, in Medellín, the use of fireworks is not a centralized event, but rather they are set off by people along and across the valley, with a tendency to be concentrated in the poorest neighborhoods. The generalized use of fireworks has made La Alborada even more unpopular given that the Aburrá Valley, home to approximately four million people, has endured the onset of critical air-quality episodes during the last 5 years as a result of significant anthropogenic emissions and adverse meteorological conditions for vertical dispersion of pollutants. The latter is associated with thin ABLs during certain seasons, as a result of regional scale forcing at the annual and interannual time scales (Herrera-Mejía and Hoyos 2019). In addition to La Alborada fireworks, there is also widespread fireworks use during New Year’s Eve (NYE) celebrations, with significantly less rejection by the community. Fireworks and firecrackers used in Colombia, in general, are made of black powder, which is made of potassium nitrate (the oxidant), charcoal (organic fuel), and sulfur (fuel) in a 75:15:10 mixture (Russell 2008; Mocella and Conkling 2019; Martín-Alberca and García-Ruiz 2014; Peña-Jiménez and Silva-Riaño 2008).

The goal of the present study is to evaluate the effects of fireworks’ use during La Alborada and NYE celebrations on PM concentration in the Aburrá Valley using information from 2015 to 2018, providing evidence to the local governments that, in addition to firework manipulation safety and the negative effects on wildlife and domestic animals, health-related issues associated with air quality also provide grounds for strictly enforcing the recurrent bans on fireworks fabrication, transportation, commercialization, distribution, use, possession, or carry, imposed by the national government (Gaviria-Uribe and Ospina-Martínez 2016), the City of Medellín (Gómez-Barrera 2016; Gaviria-Correa 2015), and the metropolitan environmental authority (Área Metropolitana del Valle de Aburrá, AMVA) (Gaviria-Correa and Elejalde-López 2015). Although from a different cultural background, strict enforcement of fireworks bans has helped to reduce the pollution peaks during the celebration of the Chinese new year, compared with cities without restrictions (Lai and Brimblecombe 2017).

Among the key differences of this work compared with other studies on the impact of fireworks in air quality is the use of multiple datasets of different nature, including records from the official air quality monitoring network, backscattering intensity (BI) retrievals from a ceilometer network, measurements from a dense low-cost PM citizen science network, and other in situ remote-sensing equipment to characterize the atmospheric conditions during La Alborada and NYE. As one of the novel aspects of our work, we assess pollution concentration near the surface and in the vertical, and we take into account the thermodynamic state of the atmosphere to better understand the residence time of pollution after it is emitted. The magnitude of the aerosol increase resulting from fireworks is highly variable from location to location; hence, using relatively sparse monitoring networks to quantify the risks could result in an underestimation of the PM increment. While the Aburrá Valley official PM network is considered dense, the hyper-dense low-cost network allows estimating in detail the probability density function (PDF) of the PM increments, a required input for accurate probabilistic health risks and econometric studies. We also consider socioeconomic aspects of the city of Medellín to assess the use of fireworks and the impact on local air quality. Section “Region of study and data” describes the geography and the socioeconomic characteristics of the region, as well as the different datasets used in this study. Section “Results” presents the results of the data analysis, highlighting the most critical areas within the metropolitan region where the effects are marked. Finally, Section ”Conclusions” presents the most important conclusions of the study.

## Region of study and data

In this work, the assessment of the effects of fireworks during La Alborada and NYE on pollutant concentration relies on in situ air-quality measurements from the official Aburrá Valley network and from a citizen science PM monitoring project, as well as on remote-sensing information from elastic ceilometers that serve as a proxy for PM concentration, allowing for the assessment of the vertical distribution of firework pollutants within the ABL. Thermodynamic profiles based on retrievals from a microwave radiometer (MWR), dynamic profiles from a radar wind profiler (RPW), rainfall derived from weather radar reflectivity and in situ gauges, and meteorological information from automatic weather stations are also used to characterize the state of the atmosphere during La Alborada and NYE from 2015 to 2018.

### Region of study: geographical context and socioeconomic information

**Fig. 1:**
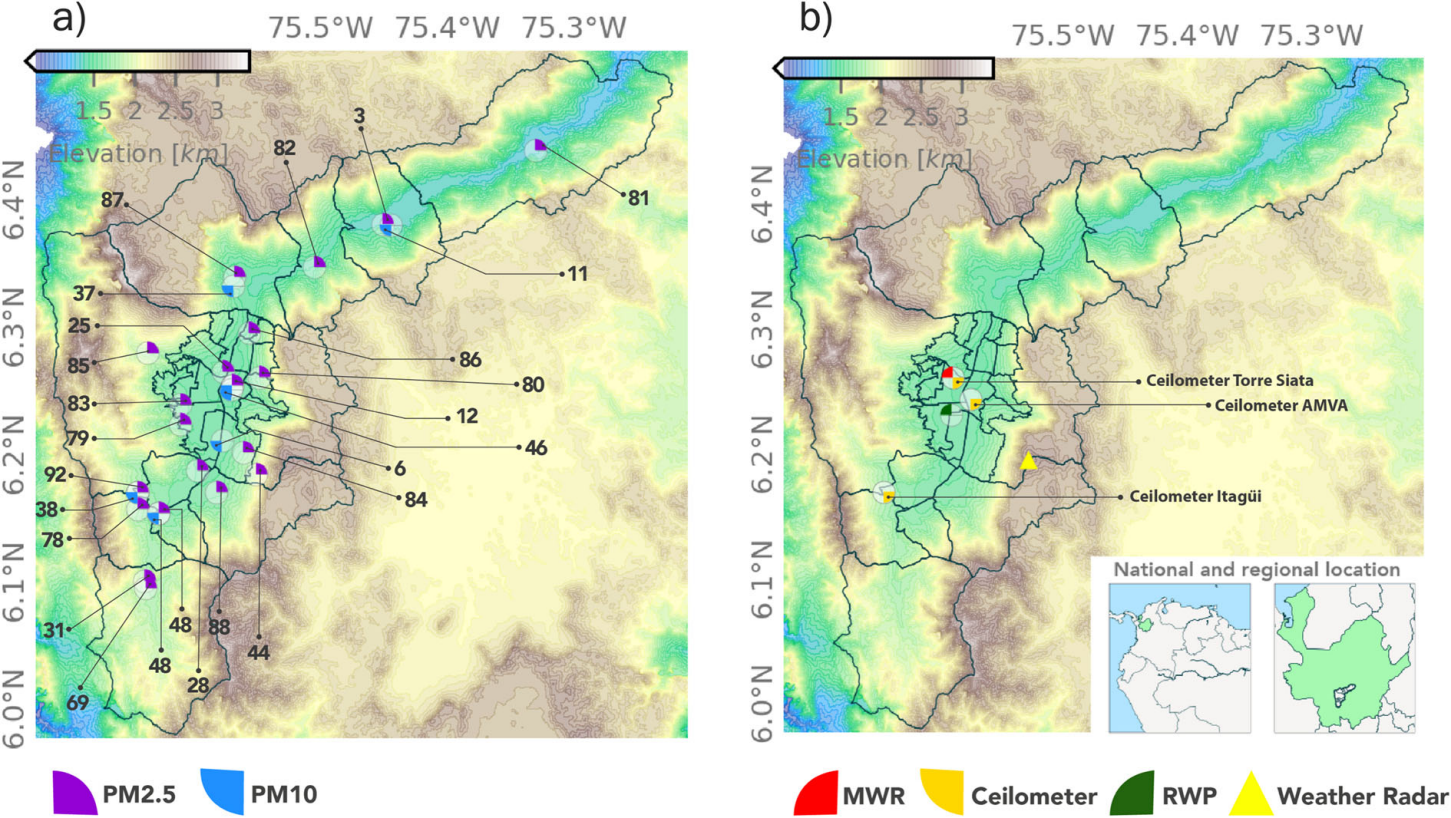
Geographical context of the Aburrá Valley, located in Colombia, Department of Antioquia. The maps shows, in brown to blue colors, the main topographic features of the region and (a) the distribution of the official air-quality monitoring stations, and (b) the microwave radiometer, the radar wind profiler, the ceilometers, and the C-Band weather radar. In this study, we use records from 21 in situ PM2.5 and 8 PM10 monitoring stations. The overall period of the air-quality records is from August 2007 to March 2018, and it is specified for each station in Table 2. PM2.5 monitoring stations 25 and 12 are closest to ceilometers at SIATA and AMVA, respectively

Figure 1 shows the geographical context of the Aburrá Valley and the location of the monitoring equipment used in this study. The valley is 64 km in length and is located in the Central Andes mountain range between 6^∘^ and 6.5^∘^ N and 75.3^∘^ and 75.6^∘^ W. The widest section of the valley, from divide to divide, is approximately 18.2 km and the narrowest section is nearly 3 km. The difference in height between the top and the bottom of the valley is nearly 1.4 km. Figure 1 also shows the political map of the Medellín metropolitan area including ten cities settled within the Aburrá Valley. The area of the metropolitan region is 1152 km^2^. Medellín, the largest and most populated city, is also the nucleus of the metropolitan area. In Colombia, the urban area in most cities, including Medellín, are administratively subdivided into communes formed by neighborhoods. Figure 1 also shows the communes of Medellín.

Regarding the socioeconomic aspects, Medellín, with a Gini index of 0.47 (Restrepo et al. 2016), a statistical measure often used to gauge the income gap between the richest and the rest of the population, indicates a region with high wealth inequality. Table 1 summarizes, for each Medellín commune, both rural and urban, the area, the population, the quality of life multidimensional index (QLMDI), and the percentage of the population with an income higher than four times the legal monthly minimum salary (Income % > 4 MMS). The socioeconomic information is obtained from the city’s planning department, as reported in Restrepo et al. (2016). The QLMDI, developed by the municipality, takes into account the housing conditions including whether the house is rented or owned, the quality of the house and the economical conditions of the neighborhood, the access to utilities, the environmental conditions, the education level of each member of the household, access to public transportation, vehicle ownership, cellular phone and domestic appliances ownership, neighborhood safety, employment conditions and income, access to health care, access to recreational activities, and perception polls (Restrepo et al. 2016). The average QLMDI for the urban areas in Medellín is 49.3 (relative arbitrary units). The most affluent communes are El Poblado and Laureles-Estadio, and the communes with the lowest average income and quality of life correspond to Popular and Santa Cruz. The latter two communes are also among the most densely populated areas in the city.

**Table 1:** Medellín’s geographical and socioeconomic information for 2018 (Restrepo et al. 2016). Rural communes do not have an official number. Values in italics in the 4th and 5th columns denote communes below the average population density (25610 persons per km^2^), and above the average QLMDI, respectively

Commune	Commune #	Area (km^2^)	Population	QLMDI	Income > 4 MMS (%)
El Poblado	14	23	*131486*	*76.6*	35
Laureles-Estadio	11	7.42	*122744*	*68.6*	28
La América	12	3.89	*96918*	*61.9*	16
Belén	16	8.83	*197399*	*57.4*	15
La Candelaria	10	7.35	*85658*	*56.4*	10
Guayabal	15	7.60	*95397*	*52.3*	6
Buenos Aires	9	5.99	*137255*	*49.9*	5
Castilla	5	6.09	*150881*	48.1	4
Robledo	7	9.38	*174496*	46.1	4
Aranjuez	4	4.87	162915	44.1	3
Doce de Octubre	6	3.84	194787	40.8	2
San Javier	13	7.42	*139175*	40.4	2
Villa Hermosa	8	5.77	138542	39.7	2
Manrique	3	5.49	161070	37.5	1
Santa Cruz	2	2.21	112514	37.1	1
Popular	1	3.09	131445	34.8	0
San Antonio de Prado	NA	60.4	117594	45.7	NR
San Elena	NA	70.46	19559	45.6	NR
Altavista	NA	27.41	38564	39.4	NR
Palmitas	NA	49.54	7061	39.2	NR
San Cristobal	NA	44.54	93072	38.8	NR

### Official air-quality monitoring network

PM concentration in the Aburrá Valley is routinely monitored by the local early warning system (Sistema de Alerta Temprana de Medellín y el Valle de Aburrá -SIATA-, www.siata.gov.co), a project of AMVA. Figure 1 a shows the spatial distribution of the automatic, real-time air-quality monitoring network instruments used in this research, including 21 PM2.5 and 8 PM10 sensors. Most PM monitoring stations are located in residential areas; three PM2.5 (stations 12, 31, and 48) and four PM10 stations (stations 12, 46, 48, and 92) are directly influenced by traffic emissions. The PM2.5 and PM10 equipment corresponds to Met One Instruments BAM-1020 and BAM-1022 monitors using a beta ray attenuation method to automatically measure airborne PM concentration levels in μgm^−3^. The official PM information from BAM-1020 and 1022 sensors is available at an hourly resolution. A Met One Instruments E-BAM sensor, also based on the beta attenuation method and a size selective second downstream sampling inlet, is used to measure PM1 at monitoring station 48 (see Fig. 1a and Table 2). A Magee Scientific AE33 Aethalometer is used for real-time measurements of aerosol BC, based on a patented DualSpot^TM^ technology and a multi-wavelength optical analysis. The AE33 aethalometer uses seven optical wavelengths from the near-infrared (950 nm) to the near-ultraviolet (370 nm), and the illumination and analysis is performed at a 1-Hz time step. The AMVA real-time air-quality monitoring network incorporates strict control tests for quality assurance.

**Table 2:** Automatic air-quality monitoring network

Station	Type	Initial date	End date	Municipality(/Commune)
3	PM2.5	2015-09-12	2019-01-29	Girardota
6	PM10	2007-08-11	2019-01-31	Medellín/Poblado
11	PM10	2008-04-03	2019-01-31	Girardota
12	PM2.5	2012-11-28	2019-01-31	Medellín/La Candelaria
	PM10	2015-09-10	2019-01-31	
25	PM2.5	2012-09-19	2019-01-31	Medellín/Robledo
28	PM2.5	2012-03-01	2019-01-31	Itagüí
31	PM2.5	2012-10-27	2019-01-31	Caldas
37	PM10	2012-11-28	2019-01-31	Bello
38	PM2.5	2012-06-06	2019-01-31	Itagüí
	PM10	2012-06-05	2019-01-31	
44	PM2.5	2015-09-16	2019-01-31	Medellín/Poblado
46	PM10	2007-08-23	2019-01-31	Medellín/La Candelaria
48	PM2.5	2014-02-04	2019-01-31	Sabaneta
	PM10	2018-04-11	2019-01-31	
	PM1	2018-04-10	2019-01-31	
	BC	2018-06-21	2019-01-31	
69	PM2.5	2018-02-24	2019-01-31	Caldas
74	PM10	2017-01-19	2019-01-31	Medellín/Robledo
78	PM2.5	2017-08-05	2019-01-31	La Estrella
79	PM2.5	2017-08-26	2019-01-31	Medellín/Belén
80	PM2.5	2017-09-05	2019-01-31	Medellín/Villa Hermosa
81	PM2.5	2017-09-19	2019-01-31	Barbosa
82	PM2.5	2017-09-26	2019-01-31	Copacabana
83	PM2.5	2017-10-03	2019-01-31	Medellín/Belén
84	PM2.5	2017-10-03	2019-01-31	Medellín/Poblado
85	PM2.5	2017-10-04	2019-01-31	Medellín/San Cristobal
86	PM2.5	2017-10-05	2019-01-31	Medellín/Aranjuez
87	PM2.5	2017-10-31	2019-01-31	Bello
88	PM2.5	2017-11-03	2019-01-31	Envigado
90	PM2.5	2018-03-19	2019-01-31	Sabaneta
92	PM10	2018-06-23	2019-01-31	Itagüí

### Citizen science network

**Fig. 2:**
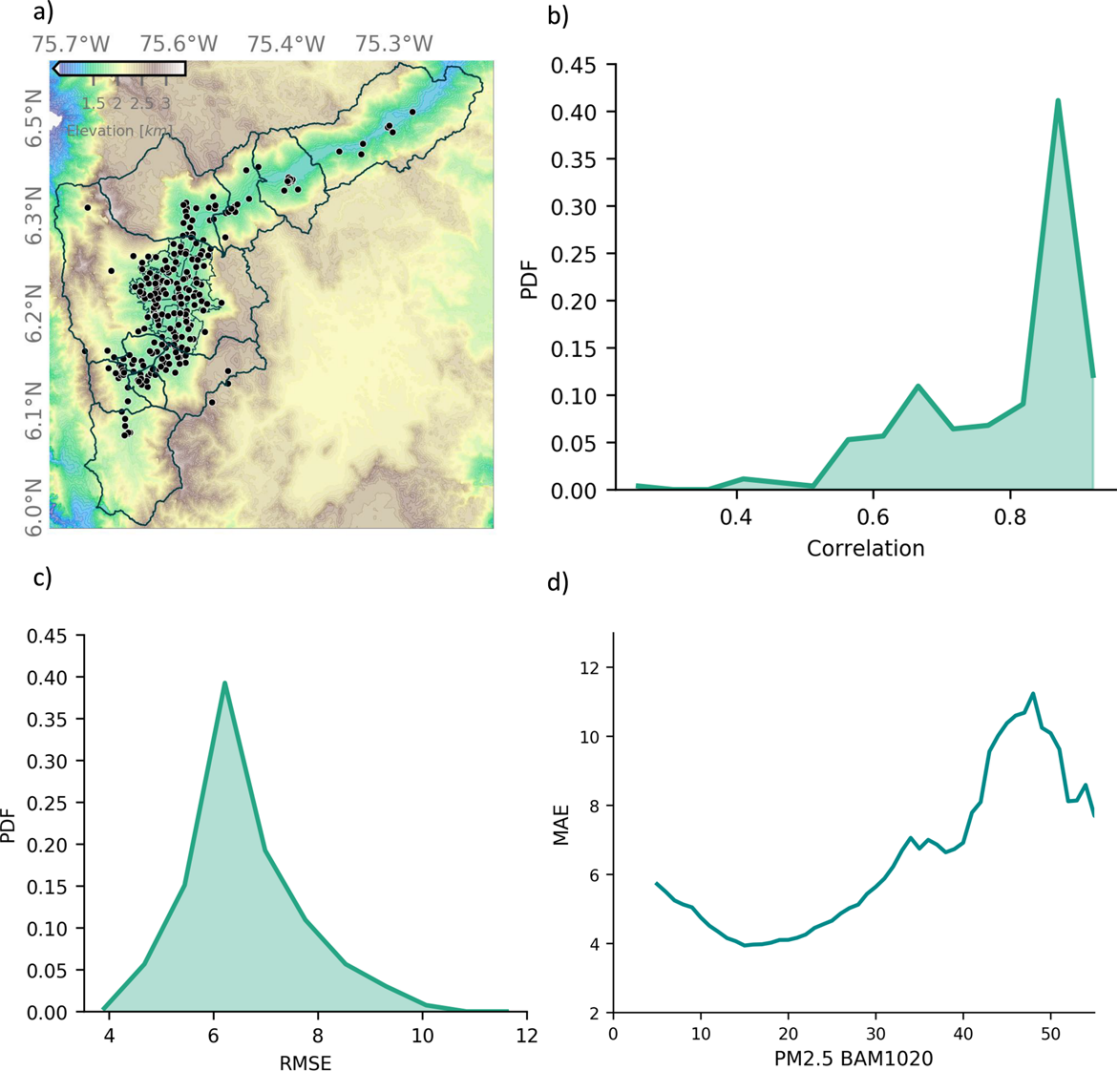
**a** Spatial distribution of 250 low-cost PM sensors from an air-quality citizen science project. **b** PDF of the Pearson correlation coefficient between the recorded PM2.5 time series using the reference BAM-1020 sensor and the collocated low-cost sensor. **c** Similar to b) for theroot mean squared error. **d** Mean absolute error (MAE) as a function of average concentration of PM2.5

Citizens are becoming increasingly involved in scientific research and environmental monitoring, actively helping the advancement of the scientific knowledge (Silvertown 2009; Conrad and Hilchey 2011; Bonney et al. 2014). In the case of air quality, citizen science projects have allowed the expansion of monitoring networks in areas with significant pollution problems, providing a higher spatial and temporal coverage compared with that of traditional monitoring methods (Holstius et al. 2014; Castell et al. 2017; Schneider et al. 2017; Austen 2015). AMVA implemented a scientific and social project known locally as *Ciudadanos Científicos* (Citizen Scientists) with the purpose of engaging the community in environmental issues and expanding air-quality monitoring. Through this initiative, by December 2018, there were already 250 real-time PM sensors installed in houses of citizen scientists. In this project, citizens measure, among other variables, PM2.5 using a monitoring equipment developed by SIATA engineers based on the low-cost Shinyei PPD42NS, NOVA SDS011, and Bjhike HK-A5 sensors. Low-cost air-quality sensors are useful for monitoring the spatio-temporal variability of particle concentration with a 1-min temporal resolution. Figure 2 a shows the spatial distribution of the 250 low-cost PM sensors from the air-quality citizen science project. Each citizen scientist monitor is calibrated individually using BAM-1020 measurements as a reference. All low-cost sensors are collocated with the reference sensors for a minimum 3-week period, collecting hourly data. To assess the overall performance of the 250 low-cost sensors, we calculate the Pearson correlation, the root mean square error, and the mean absolute error for each of the sensors, comparing the recorded PM2.5 time series from both the reference BAM-1020 sensor and the low-cost sensor, after the calibration. Figure 2b shows the PDF of the Pearson correlation coefficient indicating that most of the low-cost sensor records (91%) show a correlation above 0.6 and a high percentage of the sensors (67%) show a correlation above 0.8. Similarly, the PDF of the root mean squared error shows a median value of 6.2 μgm^−3^ (Fig. 2c). Figure 2d shows the mean absolute error (MAE) as a function of average concentration of PM2.5, suggesting that the percentage of the errors associated with the low-cost sensors decreases for high concentrations. Overall, the correlation coefficient and the error analysis indicate that the low-cost sensors represent satisfactorily well the temporal variability and the magnitude of the PM2.5 concentrations in the region. In this study, we only use low-cost sensors with a correlation coefficient of 0.7 or higher (threshold determined ad hoc).

### Ceilometers

Ceilometers provide information regarding the laser-pulse energy that is backscattered by clouds and other atmospheric components, including PM, expressed as the backscattering attenuated coefficient (*β*) (Emeis et al. 2009; Münkel and Roininen 2010; Kambezidis et al. 2013)s. Lidars and ceilometers have been widely used to detect sand to characterize aerosols in the atmosphere (Muenkel et al. 2004; Morille et al. 2007; Wiegner et al. 2014), including PM from fireworks plumes (Calhoun et al. 2004; van der Kamp et al. 2008; Han et al. 2014). For example, van der Kamp et al. (2008) show evidence of an elevated aerosol plume using ceilometer retrievals, corresponding to a pyrotechnic competition in Vancouver, Canada. In Wuhan, China, the vertical distribution of aerosols emitted by firecrackers on the ground was reported to spread to a height of more than 450 m in the atmosphere (Han et al. 2014).

Retrievals from three Vaisala CL51 ceilometers (910-nm wavelength), installed at different locations inside the valley (see Fig. 1b), are used to assess changes in the vertical BI profile as a result of fireworks use. Ceilometer BI profiles are also useful to detect the height of the ABL, based on the assumption that a significant aerosol concentration reduction occurs at the top of the latter (Hayden et al. 1997; Steyn et al. 1999; Muenkel et al. 2004; Granados-Munoz et al. 2012; Emeis et al. 2012; Stachlewska et al. 2012; Herrera-Mejía and Hoyos 2019). During fair-weather days, BI profiles often provide a good depiction of the ABL evolution within the valley. Typically, the onset of the convective boundary layer (CBL), associated with an unstable atmosphere, generates a considerable reduction in the ceilometer BI. Ceilometer profiles are available continuously (with some missing data periods) since November 2015 in all sites.

### Microwave radiometer

Temperature and humidity profiles retrieved using a Radiometrics MP-3000A microwave radiometer (MWR) are used to estimate potential temperature profiles to assess the stability of the lower troposphere. The MWR is located at the top of the SIATA main operation center on the valley floor (see Fig. 1b), approximately 60 m from the surface, and provides profiles of the thermodynamic state of the atmosphere with a variable vertical resolution, depending on the atmospheric layer: 50-m resolution for retrievals in the layer from the surface to 500 m above the surface, 100-m resolution from 500 m to 2 km above the surface, and 250-m resolution from 2 km to 10 km. Profiles are available every 2 min since January 2013 with periods of missing data mostly due to lightning-related damages.

### Radar wind profiler

Vertical wind profiles are used to assess the dynamic conditions of the atmosphere and the potential for dispersing pollutant from fireworks in the valley. The vertical wind profiles are obtained from a RAPTOR VAD-BL Doppler radar designed and manufactured by DeTect Inc. The radar relies on the refractive index variations caused by changes in humidity, temperature, and pressure. The Aburrá Valley wind profiler (see Fig. 1b) works at a nominal frequency of 915 MHz, reaching up to 8 km above the surface under high humidity conditions (Lau et al. 2013). The radar wind profiler (RWP) measures the wind profile in different modes with different vertical resolutions. We use two overlapping modes: in the higher resolution mode (60 m), the RWP measures the wind profile from 77 to 3500 m, and in the lower resolution mode (72 m), from 2500 to 8000 m. In this study, we only use the higher resolution mode since the ABL was never higher than 3500 m. The temporal resolution is 5 min and the information from the radar is available since January 2015.

### Precipitation information

Precipitation estimates from a weather radar are based on a technique described in Sepúlveda (2015) and Sepúlveda and Hoyos (2017) using weather radar and in situ disdrometer and rain gauge information. The technique allows the estimation of precipitation maps over the valley using retrievals from a C-band polarimetric Doppler weather radar operated by SIATA. The radar scanning strategy allows for obtaining precipitation information every 5 min at a spatial resolution of approximately 128 m using a 1^∘^ tilt plan position indicator sweep; the uncertainty associated with the quantitative precipitation estimates is relatively low in a 120-km radius from the installation site (Sepúlveda 2015).

## Results

### La Alborada and NYE PM changes

#### Evidence from official monitoring networks

**Fig. 3:**
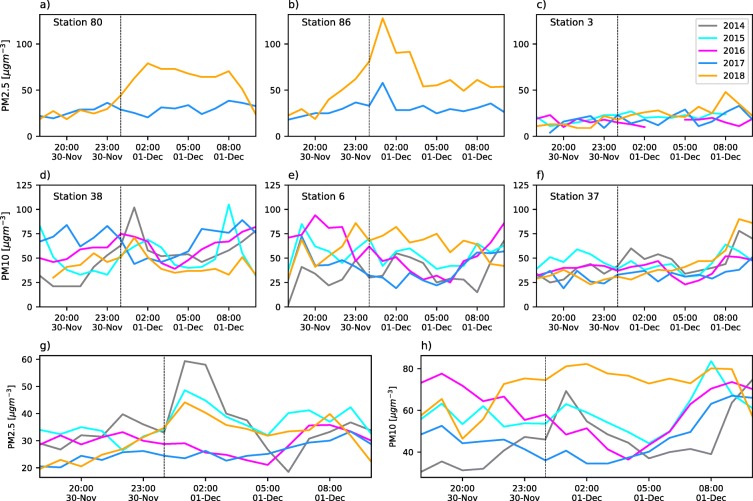
(a) to (f) Examples of hourly evolution of fine (PM2.5) and coarse (PM10) PM concentrations during the La Alborada celebration for different monitoring stations and different years from 2015 to 2018, between November 30, 18:00 LT and December 1, 10:00 LT. In this case, the monitoring stations used for PM2.5 are (a) 80, (b) 86, and (c) 3, and for PM10 (d) 38, (e) 6, and (f) 37. (g) and (h) Hourly evolution of the average concentration of PM2.5 and PM10 in the valley, using the available records, for the years from 2015 to 2018

Fireworks during the La Alborada and NYE celebrations inject a considerable amount of PM into the ABL. Figure 3a–f show examples of the hourly evolution of the typical PM2.5 and PM10 concentration for different monitoring stations and different years from 2015 to 2018, between November 30, 18:00 LT and December 1, 10:00 LT, corresponding to La Alborada. We select different monitoring stations to showcase contrasting examples, ranging from large PM concentration increments to almost no perceptible air-quality effects. The overall effect of fireworks is also presented in Fig. 3 g and h, showing the hourly evolution of the average concentration of the PM2.5 and PM10 of the valley, for each year.

Monitoring stations 80 and 86 are located in Medellín on the eastern slope, in two residential areas in densely populated communes (see commune area and population in Table 1). From a socioeconomic point of view, these two communes are below average in terms of multi-dimensional quality-of-life indicators and per capita income (see Table 1 for socioeconomic information). Stations 80 and 86 PM2.5 records are available for the 2017 and 2018 La Alborada events; station 3 records are available since 2015 (see Table 2). Station 80 (Fig. 3a) shows a tripling of PM2.5 in less than 2 h during 2018, with concentrations increasing from 25 μgm^−3^ at 23:00 LT on November 30, to 75 μgm^−3^ at 01:00 LT on December 1; station 86 presents yet a more drastic increase with a fivefold change from 25 to 125 μgm^−3^ (see Fig. 3b). On the other hand, stations 80 and 86 do not show any significant increase in PM2.5 concentration during the 2017 La Alborada, although station 86 does show a PM2.5 doubling at approximately 01:00 LT. Station 3, in the municipality of Girardota (population 54219, area 82 km^2^), does not show an important increase in any of the years since 2015 most likely because of the low population density. The average PM2.5 record considering all available sensors each year, shows a similar behavior, with the largest increase during La Alborada 2014, followed by 2015 and 2018, and no average PM2.5 increase during 2016 and 2017 (Fig. 3g).

Regarding PM10, stations 6, 37, and 38 records are available for the La Alborada events since 2014. Station 38, in a densely populated area in the city of Itagüí (population 279894, area 21.9 m^2^), shows an increase in PM10 in the range of 25 to 40 μgm^−3^ during 2015, 2016, and 2018, and a 75 μgm^−3^ increase during 2014, all associated with La Alborada fireworks (see Fig. 3d). Similar to the behavior of PM2.5, 2017 does not show an increase in PM10 concentration but rather a slight decrease. Stations 37 and 6, located within university campuses, do not show, in general, large relative PM10 increments during La Alborada events. Station 37 does show, however, a PM10 increase of 20 μgm^−3^ during 2014, coinciding with the year corresponding to the most substantial PM10 increase at station 38. Station 6 is directly influenced by traffic and it is located in Medellín in a commune with the highest socioeconomic conditions in the region (El Poblado, see Table 1), where the use of fireworks is not as widespread as in other communes. Anthropological evidence, exclusively for the City of Medellín, suggests that affluent communities tend to use less fireworks than the average family, with a preference for high-flying, colorful, pyrotechnics, while communities with less income mostly use firecrackers (Toro-Loaiza and Manrique-López 2015). The average PM10 record, considering all available sensors, also shows a similar behavior, with the largest increase during La Alborada 2014, followed by 2018, with a slight decrease during 2017, and no relevant trends during 2016 (Fig. 3h). The observed temporal evolution of the PM10 and PM2.5 concentrations during La Alborada suggests that most of the PM mass increment is a direct result of fireworks and the corresponding fine particles. It is also important to note that the fireworks-related PM dispersed faster during 2014 than during 2018, pointing to the need to assess the state of the atmosphere and its role modulating pollutant dispersion.

**Fig. 4:**
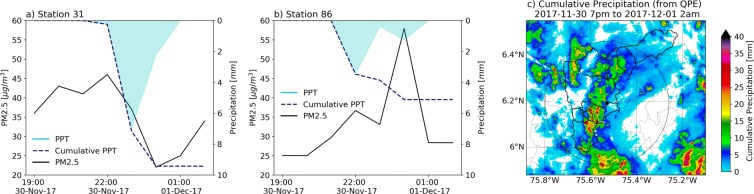
(a) and (b) Evolution of PM2.5 concentration during the 2017 La Alborada event for stations 31 (Municipality of Caldas) and 86 (Aranjuez commune, in Medellín), together with the the hourly precipitation record from each station rain gauge. (c) Cumulative precipitation during the 2017 La Alborada evening, from November 30, 2017, 19:00 LT to December 1, 2017, 02:00 LT. The spatial distribution of precipitation was obtained from C-Band radar reflectivity retrievals in combination with a disdrometer-based QPE algorithm described in Sepúlveda (2015)

The lack of an increase in PM10 and PM2.5 during 2017, as previously mentioned, is associated with a precipitation event during La Alborada, with isolated high-intensity clusters in the urban zone, in several residential areas in the Valley (Fig. 4). Precipitation has a twofold effect with respect to fireworks-related pollution: (i) precipitation discourages the widespread use of fireworks given that citizens opt to not be exposed to rainfall, particularly if the intensities are high, and (ii) precipitation in stable atmospheres generates efficient rainfall-induced below-cloud scavenging (e.g., Roldán-Henao et al., 2019). Figure 4 shows the cumulative precipitation during the 2017 La Alborada evening, from November 30, 2017, 19:00 LT to December 1, 2017, 02:00 LT, and evidence of precipitation-driven below-cloud scavenging in one of of the monitoring stations associated with cumulative rainfall higher than 5 mm. Figure 4 a shows the PM2.5 evolution during the 2017 La Alborada event for station 31 (Municipality of Caldas), together with the hourly precipitation record from the station rain gauge. At this location, precipitation lasted for approximately 3 h, from November 30, 22:00 LT to December 1, 01:00 LT with a cumulative rainfall of 9.5 mm. The concentration of PM2.5 sharply decreased during the precipitation event, with hourly values dropping from 46 to 22 μgm^−3^. After the rainfall event ended, the concentrations increased again to 35 μgm^−3^. A different case is observed at station 86, located in the Aranjuez commune, in Medellín. The rainfall in this commune was less than 5 mm in total, explaining the observed PM2.5 doubling associated with La Alborada in 2017: precipitation started around 21:00 LT, November 30, and lasted until 01:00 LT, December 1; PM2.5 concentration decreased from 22:00 to 23:00 LT as a result of the precipitation; however, the precipitation intensity decreased considerably around midnight, allowing the community to use fireworks, increasing the pollutant concentrations from November 30, 23:00 LT, to December 1, 00:00 LT. Rainfall restarted after midnight, triggering below-cloud scavenging (and a decrease in PM2.5) once again. Both cases highlight the importance of the timing of the precipitation event relative to around midnight, when most of the fireworks are set off. The overall decrease in PM10 observed in Fig. 3 h during the 2017 La Alborada is consistent with previous studies showing that the collection efficiency is minimal for particles with an aerodynamic diameter in the so-called Greenfield gap (Greenfield 1957), approximately between 50 nm and 2 μm, and higher for coarser particles (Quérel et al. 2014; Cherrier et al. 2017), resulting in a more efficient washout of PM10 than PM2.5 (Roldán-Henao et al. 2019).

**Fig. 5:**
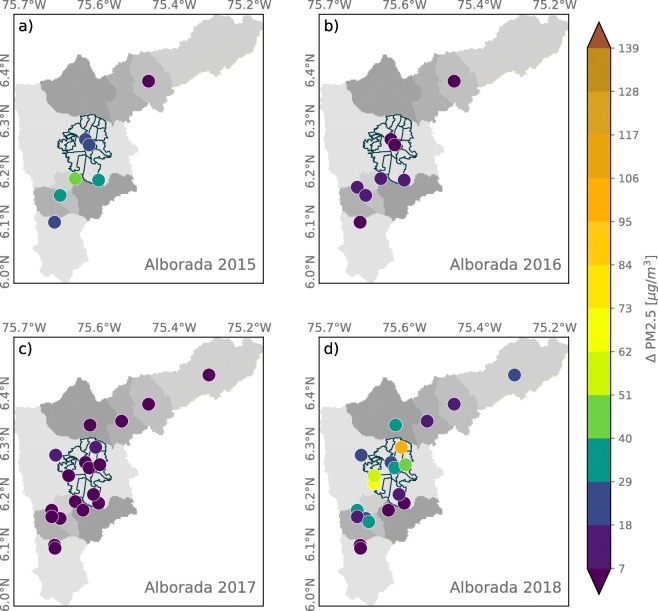
Anomaly in PM2.5 concentrations associated with La Alborada fireworks for (a) 2015, (b) 2016, (c) 2017, and (d) 2018, estimated as the difference in PM2.5 between December 1, 00:00–01:00 LT and November 30, 00:00–01:00 LT

**Fig. 6:**
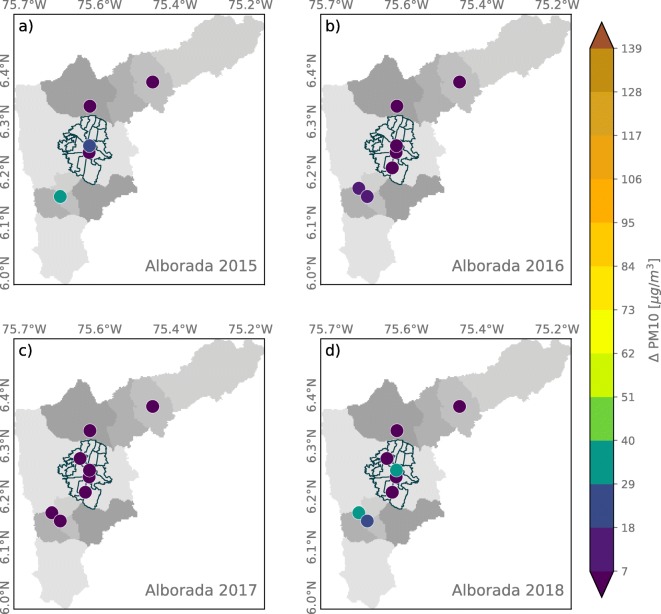
Similar to Fig. 5 for the anomaly in PM10 concentration associated with La Alborada fireworks

Figures 5 and 6 show a summary of La Alborada PM effects as the anomaly in PM2.5 (*Δ* PM2.5) and PM10 (*Δ* PM10) concentration for the years 2015 to 2018, for all stations available every year as described in Table 2, estimated as the difference between the December 1 00:00–01:00 LT concentration and that of November 30 00:00–01:00 LT. The evidence for the increase in PM2.5 as a result of fireworks use is striking given that all stations, during all years, show an increase from as low as 7 to as high as 100 μgm^−3^, with significant variability from year to year as a result of changes in the mass of firework emissions (i.e., some regions use more fireworks than others during particular years), weather-related events, and even social events. Anomalies in PM10, where available, show a similar year-to-year and spatial variability (see Fig. 6). It is important to note that, unfortunately, there are no records or estimations of the mass of black powder used each year during the celebrations. As mentioned in the introduction, fireworks are banned every year, especially since November to January; for this reason, there are no records on the mass of black powder used yearly during La Alborada and NYE.

According to Figs. 5 and 6, 2016 and 2017 show the lowest increments in PM associated with La Alborada. During 2017, as previously mentioned, a rainfall event discouraged people in the region from using fireworks after midnight and generated below-cloud scavenging, effectively removing aerosols from the lower atmosphere. In different circumstances, during 2016, the use of fireworks in the La Alborada celebrations was considerably reduced due to a tragic event that spontaneously inspired a mourning period in Medellín and most of the metropolitan area: *Chapecoense*, a small Brazilian football club, reached the *Copa Suramericana* final to be played against *Atlético Nacional 29*$\bigstar $, a team from Medellín. The plane carrying the Brazilian team to the match was en route to Colombia on November 28, 2016, when it crashed near Medellín just 11 miles from the international airport, killing 71 of the 77 people on board. The accident was reported globally by major news outlets^1^ and sparked tributes from the world of football. The people from Medellín was deeply moved by the tragedy, and as of different solidarity demonstrations with the Chapecoense club members and their families, there was a city-wide call through social media,^2^ including messages from the major and different personalities, for not using fireworks during La Alborada as a sign of respect for the peopled that perished in the accident (Duque-Suárez 2018). Most local and national news outlets reported on the success of the social campaign,^3^ also highlighting, citing sources from the public health authority of the city, that there were no injuries during the 2016 La Alborada due to manipulation of fireworks, compared with the number of injuries during La Alborada from 2012 to 2015, which had been 14, 17, 27, and 24. In 2017, the number of injuries was 5, and during 2018, there were 13 fireworks-related accidents. In contrast, 2018 was the year with the largest increments in PM2.5 concentration resulting from the La Alborada fireworks, and the spatial distribution shows the largest anomalies occurring in densely populated areas in Medellín (in the communes of Aranjuez, Belén, and Villa Hermosa).

**Fig. 7:**
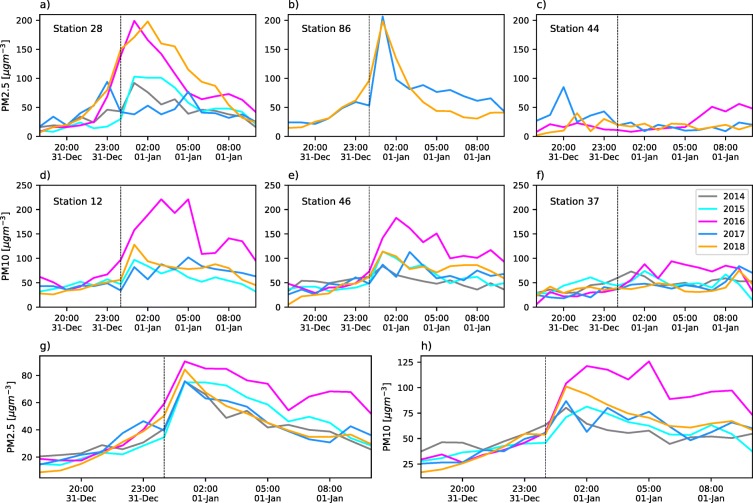
Hourly evolution of PM2.5 and PM10 concentration during NYE celebrations (between December 31, 18:00 LT and January 1, 10:00 LT.) for different monitoring stations and different years from 2014 to 2018. In this case, the monitoring stations used to showcase PM2.5 changes were numbers (a) 28, (b) 86, and (c) 44 and for PM10 numbers (d) 12, (e) 46, and (f) 37.

During NYE celebrations, the change in concentrations around midnight is more notorious than during La Alborada. Similar to Fig. 3, Fig. 7 shows examples of the hourly evolution of PM2.5 and PM10 concentrations for different monitoring stations between December 31, 18:00 LT and January 1, 10:00 LT. Figure 7 includes records from stations 12, 86, and 44 for PM2.5 and stations 28, 46, and 37 for PM10 (see Fig. 1). Station 2 is in a residential-commercial area in the city of Itagüí and station 44 is in the El Poblado commune in a low-population density residential area. Stations 28 and 86 show significant increments in PM2.5 concentration during 2018, with hourly peaks reaching up to 200 μgm^−3^, corresponding to a 30-fold increase compared with the concentration levels at approximately 18:00 LT on December 31. Station 86 also shows a considerable increase during 2017. Interestingly, the PM2.5 concentration recession after the 01:00 LT peak is slower during 2017. The recession is discussed later considering the thermodynamic state of the atmosphere. Station 28 also shows a notorious peak during 2016, with a similar magnitude to that during 2018, and two lower-magnitude increments during 2014 and 2015. Station 44, given its location, is not affected by the pollution associated with fireworks as it is clear in Fig. 7c. Stations 12 and 46, both in the downtown area, registered the largest increments in PM10 around midnight on December 31, 2016 (see Fig. 7d, e). A two- to threefold increase in PM10 was observed during the 2015 and 2018 NYE celebrations at the latter stations. Station 37 shows the least changes during NYE among all PM10 stations. In the case of NYE, the average PM2.5 record considering all available sensors each year, shows that, every year, there is at least a 3.5-fold increase in PM2.5, with the largest increase during NYE 2016, followed by 2018 (Fig. 7g). The average increase in PM10 during NYE is similar to that observed for PM10 (Fig. 7h).

**Fig. 8:**
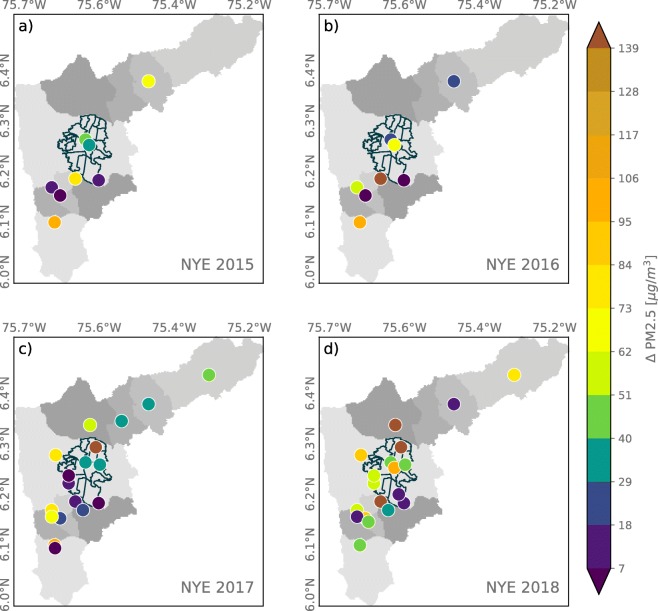
Anomaly in PM2.5 concentrations associated with NYE for (a) 2015, (b) 2016, (c) 2017, and (d) 2018, estimated as the difference in PM2.5 between January 1, 00:00–01:00 LT and December 31, 00:00–01:00 LT

**Fig. 9:**
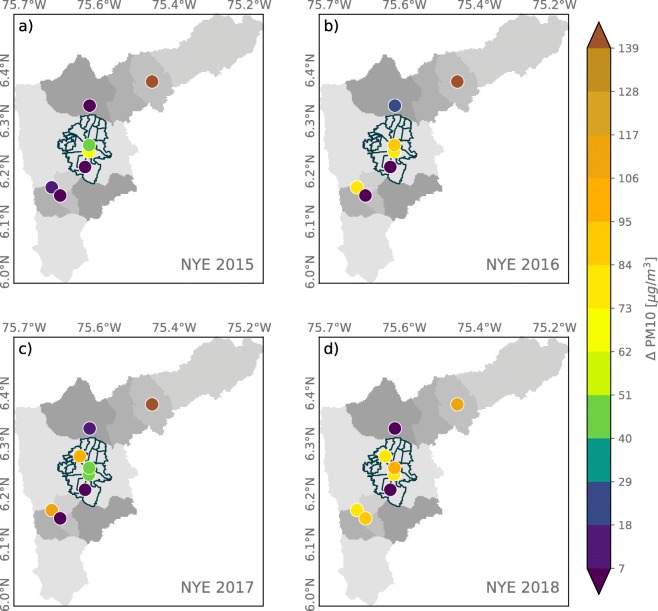
Similar to 8, the anomaly in PM10 concentrations associated with NYE celebrations

The PM2.5 and PM10 concentration anomalies associated with NYE fireworks, from 2015 to 2018, were estimated similarly as for La Alborada and are presented in Figs. 8 and 9, respectively. The evidence for all available records each year shows positive PM2.5 concentration anomalies, in some cases higher than 150 μgm^−3^. Despite the widespread educational campaigns^4^ as part of the administration strategies to reduce fireworks use, the year with the larger anomalies in PM2.5 concentration corresponds to 2018. The magnitude of the anomalies in PM10, shown in Fig. 9, is similar to that of PM2.5. Similar to the evidence for La Alborada, this suggests that the main effect of fireworks on aerosol concentration is to increase the percentage of fine PM. The observed midnight PM changes in the Aburrá Valley associated with fireworks are greater than the increments reported in most studies throughout the world (Vecchi et al. 2008; Tsai et al. 2012; Seidel and Birnbaum 2015), except those reported for the Diwali festival, in India (Barman et al. 2008; Yerramsetti et al. 2013).

#### PM10, PM2.5, PM1, and BC

**Fig. 10:**
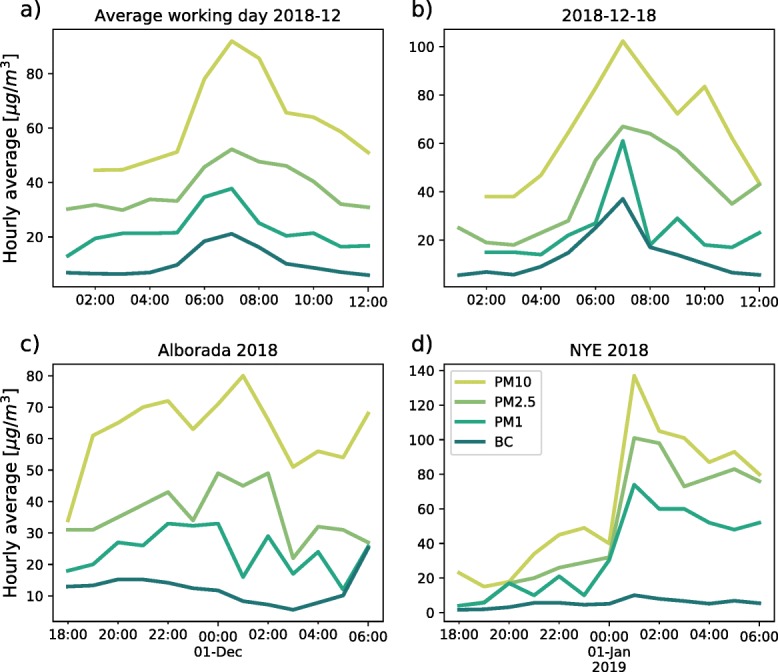
(a) Average hourly evolution of PM10, PM2.5, BC, and PM1 concentrations at station 48 for the month of December 2018. (b) Similar to (a) for the morning of December 18, 2018. (c) and (d) Similar to b) for the evening of November 30 and December 31, 2018, respectively, corresponding to La Alborada and NYE fireworks emissions.

The monitoring equipment in station 48, generally affected by direct traffic emissions, allow for assessing the temporal evolution of PM10, PM2.5, PM1, and BC concentration at the same site, as shown in Fig. 10. The figure shows the average hourly evolution of PM10, PM2.5, BC, and PM1 concentrations at station 48 for the month of December 2018, during the morning hours (Fig. 10a), as well as the evolution of all four variables for a typical working day (December 18, 2018) compared with that during La Alborada and NYE (Fig. 10b–d).

The average hourly evolution of PM10, PM2.5, BC, and PM1 for December 2018, and the records for the morning of December 18, 2018, show evidence of environmental pollution due to high vehicular emissions, with a peak in PM concentration around 07:00 LT, with a large increase in PM1 and BC concentrations. Figure 10a and b suggest a large increase in the proportion of PM2.5, with the relative average concentration of PM1 changing from 35% (45% for December 18, 2018) at around 03:00 LT to 62% (80%) during the rush hour. The net increment in PM10 is, in average, approximately 50 μgm^−3^, of which 20 μgm^−3^ corresponds to the increase in PM1, 14 μgm^−3^ corresponds to the increase in particles larger than 1 μm and smaller than 2.5 μm, and 16 μgm^−3^ corresponds to the increment in particles larger than 2.5 μm and smaller than 10 μm. In other words, 7 out of each 10 μgm^−3^ increment is associated with the increase in ultrafine and fine PM. The BC also increases, in average, from 16% of the total PM10 at around 03:00 LT to approximately 23% at 07:00 LT.

The records during the evenings of November 30, and December 31, 2018, corresponding to La Alborada and NYE fireworks emissions, show a different behavior compared with a regular day, with almost no increase in the net amount of BC in the atmosphere, hence, a decrease in the relative proportion out of the total PM10, from 20 to 25% at 11:00 LT to approximately 10% at 01:00 LT, during both days. This result is different from the evidence presented in Yerramsetti et al. (2013) and Pathak et al. (2015) showing an increase in BC due to firework-related emissions. The relative proportion of ultrafine PM, out of the total PM10, remains relatively constant during La Alborada and NYE at about 45% of the mass. During NYE, 2018, the total increase in PM10 at station 48 is 80 μgm^−3^, out of which, 50 μgm^−3^ (62%) corresponds to PM1, 25 μgm^−3^ (31%) to particles larger than 1 μm and smaller than 2.5 μm, and 5 μgm^−3^ (7%) to particles larger than 2.5 μm and smaller than 10 μm. Another difference between fireworks-induced PM increments and those resulting from direct combustion engine vehicle emissions is the mass percentage of ultrafine particles; in addition to a higher BC content, fossil-fuel-powered vehicles generate higher ultrafine particle content compared with that of fireworks.

#### Evidence from the citizen science network

**Fig. 11:**
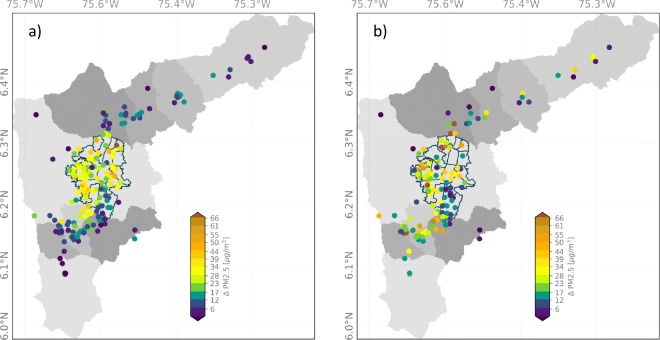
Spatial distribution of the PM2.5 concentration anomalies (*ΔPM*2.5) associated with the 2018 **a** La Alborada and **b** New Year’s Eve celebrations from the citizen scientists low-cost network records

In addition to the official air-quality network, the low-cost PM citizen science monitoring network also provides evidence of marked changes in air pollution following the La Alborada and NYE celebrations. The high density of low-cost monitors allows identifying the communes and neighborhoods with the largest PM increments after midnight. Figure 11 shows the spatial distribution of the PM2.5 concentration anomalies (Δ*PM*2.5) associated with the 2018 La Alborada and NYE celebrations, using the records from citizen scientists network. During La Alborada, the largest PM changes are confined to Medellín, in particular to the eastern hill communes, with the exception of El Poblado (southeasternmost Medellín commune). The northeastern Medellín communes show an average increase larger than 42 μgm^−3^, while the western communes show an average increase of nearly 27 μgm^−3^. The increase during NYE is larger than that during La Alborada, and it is observed not only in Medellín but also in the urban area of entire metropolitan area, mainly to the south of Medellín (Itagüí, Envigado, and Sabaneta), where the municipalities are in conurbation.

**Fig. 12:**
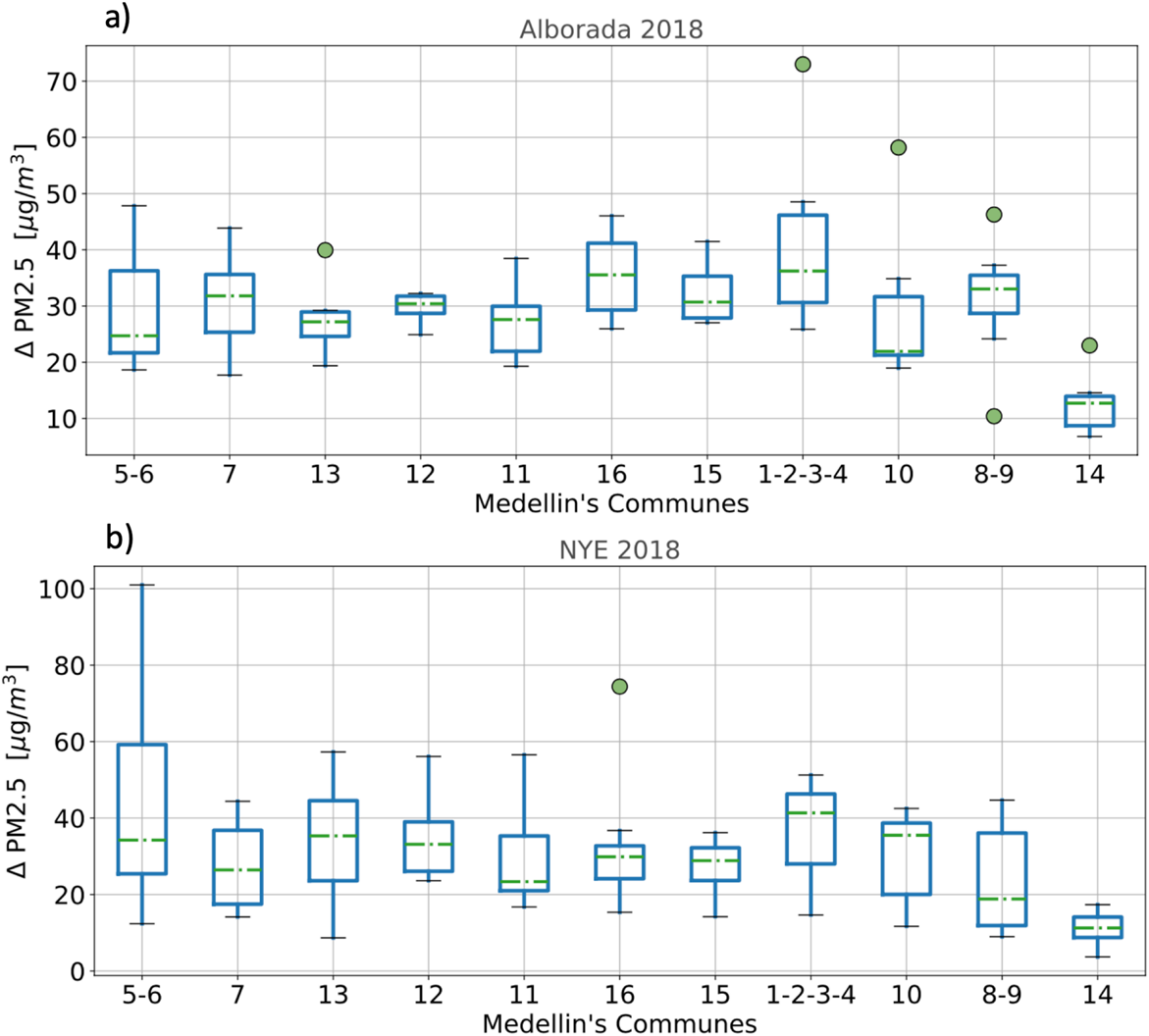
Summary of the PM2.5 concentration anomalies during (a) La Alborada and (b) NYE, by commune (or group of communes as indicated). The box-and-whisker plots depict the quartiles for each region, with the box extending the range of the outer values, and the horizontal corresponding to median. The whiskers show the range of the data computed as 1.5 times the inter-quartile range (IQR = Q3 - Q1). Points correspond to outliers. The communes are organized from west (left) to east (right)

The lowest positive concentration anomalies during the 2018 La Alborada and NYE celebrations correspond to El Poblado, which at the same time is the more affluent commune and has the lowest population density (see Table 1). During La Alborada and NYE (See Fig. 12), communes 1–4 show the highest air contamination due to fireworks (highest median value). These communes are below the average quality-of-life index and are above the average population density. Most of the features shown in Fig. 12 can be associated with population density (the higher the density, the higher the PM2.5 anomalies), quality of life (the lower the quality of life, the higher the PM2.5 anomalies), and hill location. During La Alborada, the PM2.5 anomalies increase from west to east, and, conversely, during NYE anomalies, increase from east to west. The latter difference is likely a result of intravalley pollution advection.

**Fig. 13:**
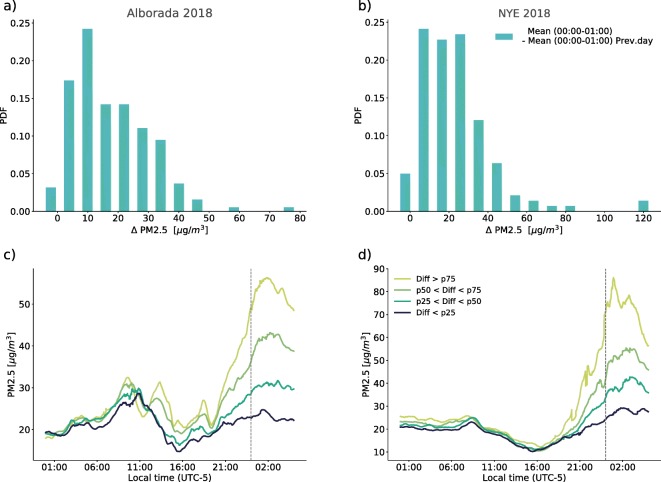
Histogram of PM2.5 concentration (*Δ* PM2.5) anomalies after midnight associated with the 2018 (a) La Alborada and (b) NYE celebrations from the citizen scientists low-cost network. (c), (d) Temporal evolution of the average PM2.5 concentration around La Alborada and NYE (dashed vertical lines), respectively, for (i) low-cost monitoring stations reporting an anomaly greater than *q*_75_, (ii) stations reporting an anomaly greater than *q*_50_ and lower than *q*_75_, (iii) stations reporting an anomaly greater than *q*_25_ and lower than *q*_50_, and stations reporting an anomaly lower than *q*_25_. Note that (a–d) have different horizontal (vertical) scales for *Δ* PM2.5

Figure 13a and b show a comparison of the histogram of the PM2.5 concentration anomalies (*Δ* PM2.5) after midnight associated with the 2018 La Alborada and NYE celebrations using measurements from the citizen scientists network.The values of the *Δ* PM2.5 quartiles, *q*_25_, *q*_50_, and *q*_75_, are, for La Alborada, 7.8, 14.9, and 27.1 μgm^−3^, respectively, and for NYE, 10.2, 20.0, and 30.3 μgm^−3^. The magnitude of the quartiles was used to summarize the temporal evolution and PM2.5 magnitude changes registered by the low-cost sensors. Figure 13c and d show the temporal evolution of the average PM2.5 concentration around La Alborada and NYE, respectively, for every *Δ* PM2.5 quartile. On average, 25% of stations in the valley show an increment, from 22:00 LT to the peak concentrations (generally between 01:00 and 02:00 LT of the following day), of 25 μgm^−3^ (from 35 to 60 μgm^−3^) during La Alborada, and of 40 μgm^−3^ (from 40 to 80 μgm^−3^) during NYE. The median increments relative to 22:00 LT concentrations, which correspond to a representative value for the entire valley, are 15 and 20 μgm^−3^ during La Alborada and NYE, respectively. Figure 13c and d also show the considerably larger magnitude of the fireworks-induced PM peaks relative to the rush hour PM2.5 peak around 07:00–9:00 LT.

### Aerosol vertical profile

The BI from two ceilometers installed on the base of the Aburrá Valley is used to study the vertical structure of the aerosol plume from fireworks during La Alborada and NYE. BI is a proxy for aerosol concentration; large values of BI, excluding those resulting from the presence of clouds, are usually associated with large aerosol loads. Also, atmospheric humidity accentuates the size of hygroscopic particles, enhancing the lower-troposphere ceilometer BI signal (Emeis et al. 2012; Young and Whiteman 2015). As previously mentioned, BI profiles generally depict the marked diurnal cycle of the ABL height. Around midnight, the ABL height at the base of the Aburrá Valley is typically close to 500 m, limiting the available control volume for the pollutants to disperse (Herrera-Mejía and Hoyos 2019). By the time the CBL starts to develop due to the onset of atmospheric instability, around 10:00 LT, the concentration of aerosols near the surface and within the valley decreases.

**Fig. 14:**
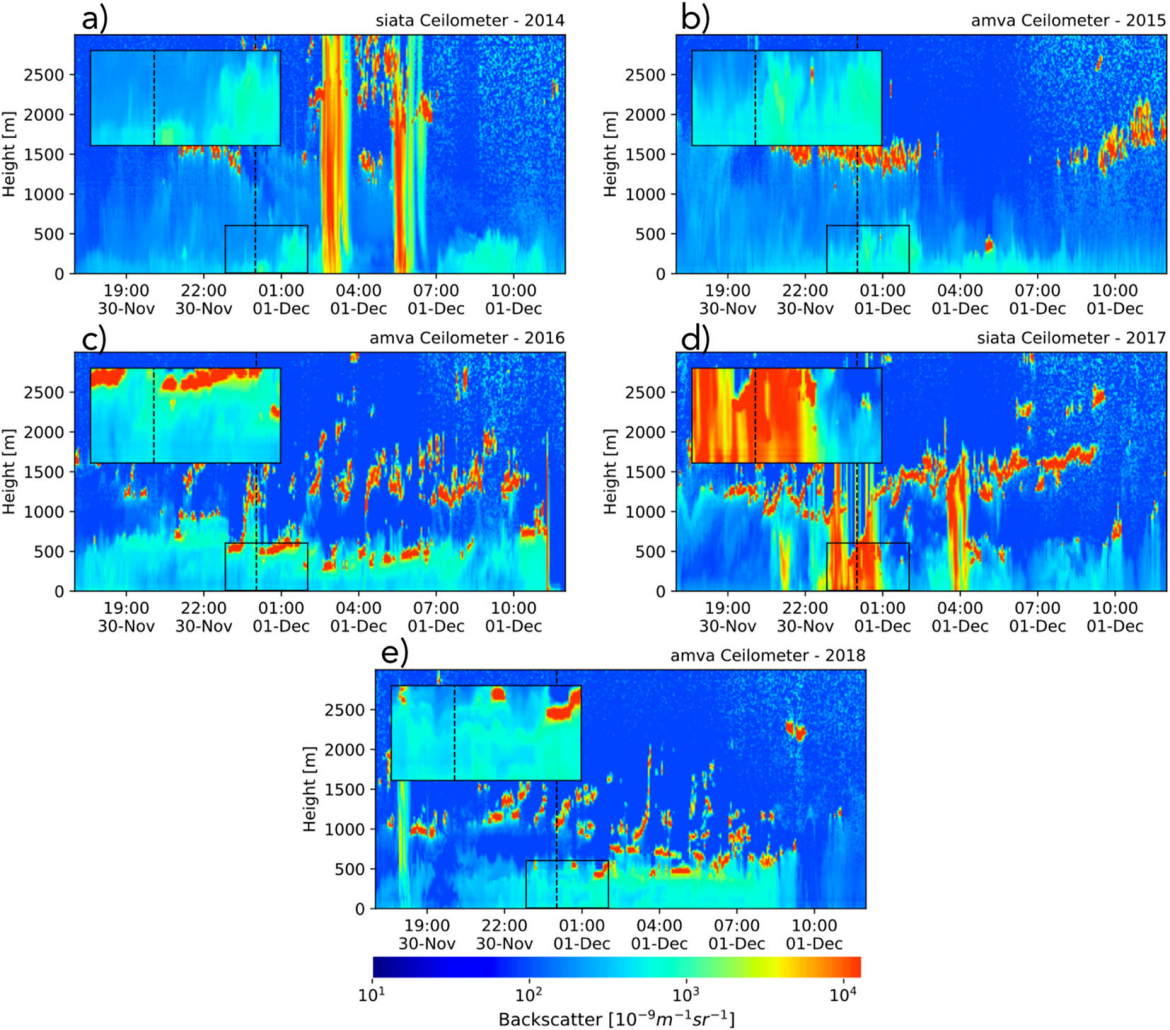
Lower troposphere (0–3000 m above the surface) ceilometer BI profiles for La Alborada cases (2014 to 2018) as retrieved at the SIATA (a, d) and AMVA (b, c, e) sites. The time domain spans from November 30, 17:00 LT, to December 1, 12:00 LT, to highlight the changes in aerosol concentration and vertical distribution during the La Alborada transition. Colors represent BI measured by Vaisala-CL51 ceilometers near the center-bottom of the valley. The insert shows a zoom of the BI around December 1st, 00:00 LT, for the first 500 m above the surface. AMVA and SIATA ceilometers are located less than 3 km apart from each other, at the base of the valley. Both ceilometers show a very similar temporal evolution of the ABL and BI profiles. We use different stations to make sure there were no missing data around midnight and before the development of the convective layer in order to show, each year, the complete evolution of the ABL

**Fig. 15:**
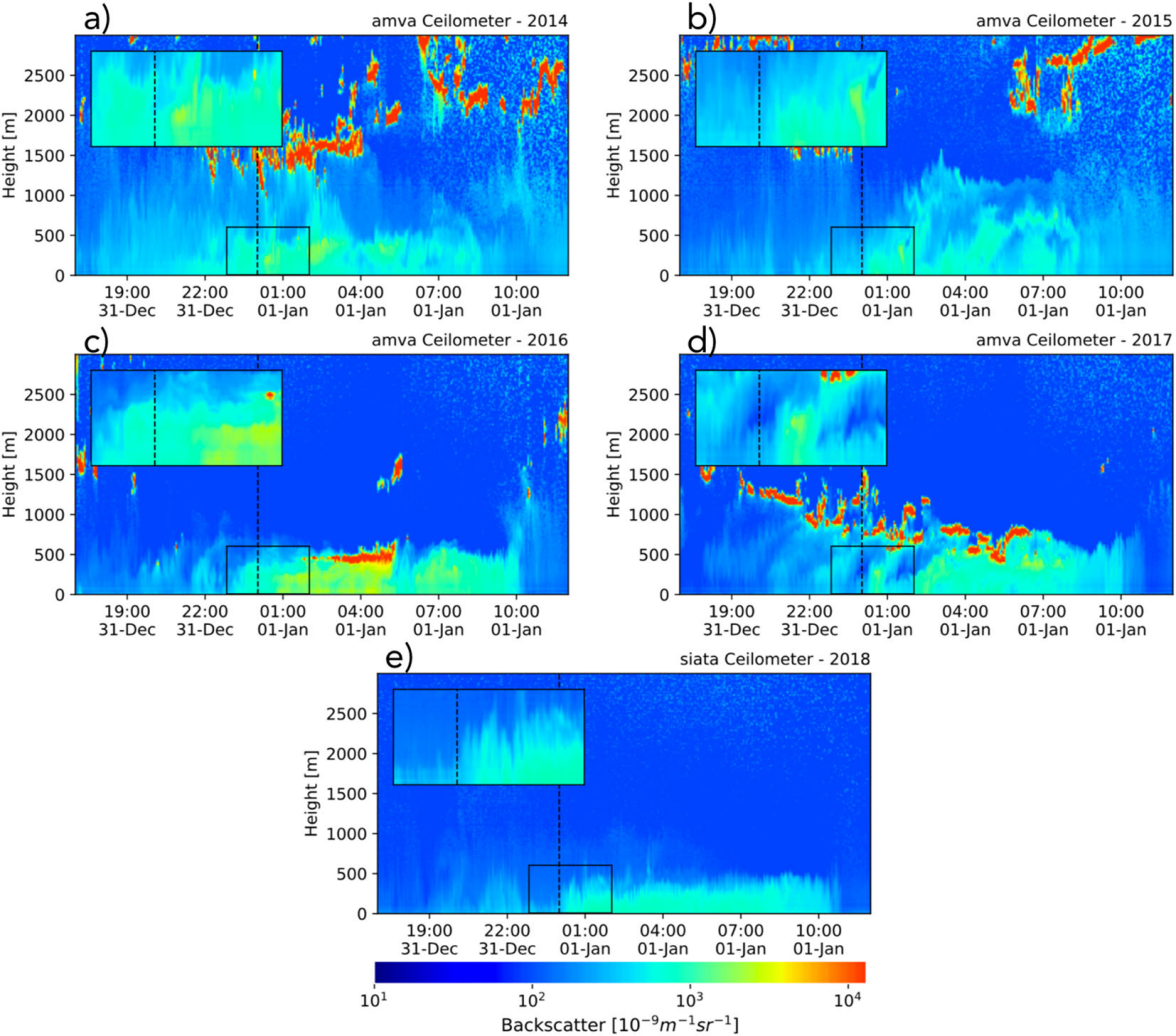
Similar to Fig. 14 for NYE. The time domain spans from December 31, 17:00 LT, to January 1, 12:00 LT, highlighting the changes in aerosol concentration and vertical distribution during the NYE transition. The insert shows a zoom of BI around January 1, 00:00 LT, for the first 500 m above the surface

Figures 14 and 15 show ceilometer retrievals, from 2014 to 2018, showing the temporal evolution of BI before, during, and after La Alborada and NYE midnight, respectively. The figures show the lower troposphere (0–3000 m above the surface) BI profiles and also a zoom of BI around La Alborada and NYE midnights, for the first 500 m. Figure 14 a presents the 2014 La Alborada case, showing a substantial change in the BI immediately after midnight, first confined to the first 100 m from the surface and then extending to the entire ABL, up to 550 m, around December 1, 2014, 01:00 LT. The evidence suggests that sufficient aerosols are emitted because of the use of fireworks, quickly replenishing the control volume confined by the surface and the first atmospheric capping inversion (ABL height), causing a noticeable discontinuity in the BI signal. Around 03:00 LT, a precipitation event, the first after midnight (evident in the high BI values—red colors), caused important below-cloud scavenging, effectively reducing BI retrievals associated with aerosols from fireworks after the rainfall ceased. For 2015 (Fig. 14b), the evidence is very similar to that for 2014, with a sudden BI increase after midnight, up to approximately 550 m. The years 2016 (Fig. 14c) and 2017 (Fig. 14e) do not present BI increases during the La Alborada celebration, matching the air-quality in situ observations shown in the previous subsections, with 2016 corresponding to the Chapecoense tragedy and 2017 to a rainfall event discouraging the use of fireworks and generating below-cloud scavenging. During 2018 (Fig. 14d), there is also a sudden BI increase after midnight, the largest for La Alborada, and up to 500 m; the high values of BI lasted, in this case, until 09:00 LT, when most likely the atmosphere became unstable as it is shown in the following section based on thermodynamic profiles from the MWR. The nearest PM2.5 monitoring station (station 25, see Fig. 1) shows a very similar behavior to the one shown by the ceilometers near-surface, with peak hourly concentrations of 70, 43, 30, 33, and 84 μgm^−3^, during La Alborada 2014, 2015, 2016, 2017, and 2018, respectively.

Cloudiness and rainfall are less likely during NYE than during La Alborada: NYE is during one of the two dry seasons in the Aburrá Valley, while La Alborada is during one of the wet-to-dry transitional seasons. Figure 15 presents the NYE cases from 2014 to 2018, showing a marked increment in BI immediately after midnight during all years, with the most prominent peaks during 2016 and 2018. The BI increase is fairly homogeneous within the ABL and it is prolonged in time; this feature is different from previous reports, using ceilometers, of fireworks plumes that are localized in time and height (Calhoun et al. 2004; van der Kamp et al. 2008). The latter is most likely because, as previously mentioned, fireworks are not part of a centralized event, but their use is widespread in most communes. The vertical distribution of aerosols from fireworks is similar to the reports for Wuhan, China (Han et al. 2014). None of the cases are modulated by rainfall; however, cloudiness does vary from year-to-year, with 2014 and 2017 corresponding to cloudy conditions during NYE and 2016 and 2018 to cloud-free conditions during the night. Aerosols linked to fireworks use quickly disperse within the ABL, occupying the entire control volume. The most salient difference among the years is that, in most cases, the aerosol vertical distribution presents evidence of a marked and steady capping inversion, persistent throughout the night (e.g., during 2016 and 2018), while during other years (e.g., 2014 and 2015), aerosol plumes seem to vertically penetrate above 500 m. This is likely associated with the stability of the lower layers, and it is explored in the following section. As during La Alborada, the nearest PM2.5 monitoring station shows a very similar behavior to ceilometers BI near-surface, with peak hourly concentrations of 61, 63, 160, and 110 μgm^−3^, during La Alborada 2014, 2015, 2016, and 2018, respectively (PM2.5 at station 25 is not available for the 2017 NYE). Note that the largest BI near the surface is observed during 2016, matching the PM2.5 records.

### Atmospheric stability

**Fig. 16:**
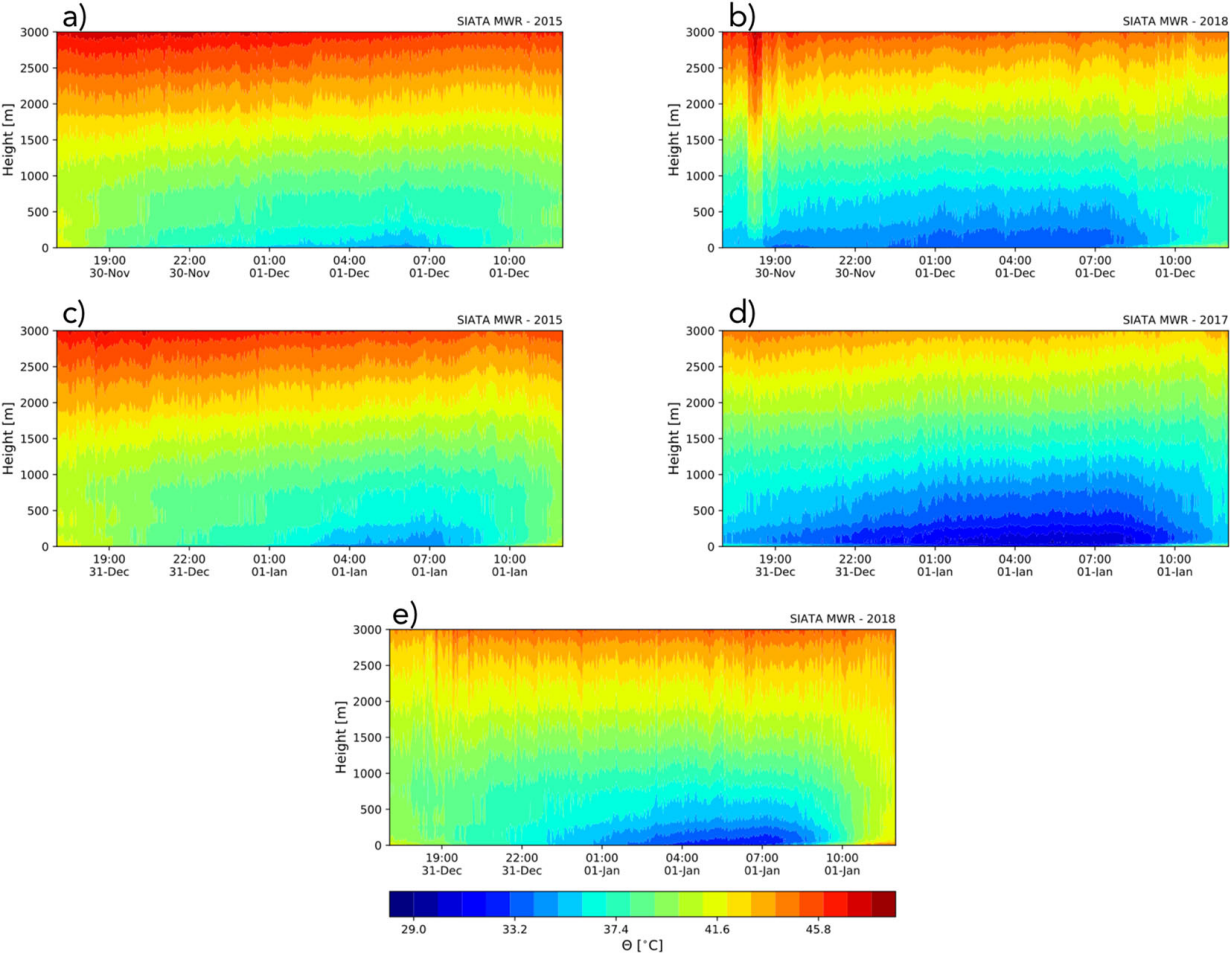
Evolution of the potential temperature vertical profile from the surface to 3000 m before, during, and after midnight (from 17:00 LT to noon) for two La Alborada (panels a and b) and three NYE (panels c to d) cases

The magnitude and the prolongation of the near-surface atmospheric stability determine the height of the stable layer (Stull 1988), where aerosols remain before the onset of the CBL. Once the CBL is established, the aerosols vertically disperse, reducing the concentration at the surface. The vertical gradient of the potential temperature determines the magnitude of the stability and establishes the required sensible heat flux to offset the stability (Leukauf et al. 2015). Figure 16 shows the evolution of the vertical profile of the potential temperature from the surface to a 3000-m height before, during, and after midnight (from 17:00 LT during the evening corresponding to the celebration to noon the following day) for La Alborada (Fig. 16a and b) and NYE (Fig. 16c–e). All the panels show a thermal inversion during this period, acting as a precursor of the shallow stable layer. A more pronounced thermal inversion leads to a slower dispersion and recession of the aerosol concentration.

**Fig. 17:**
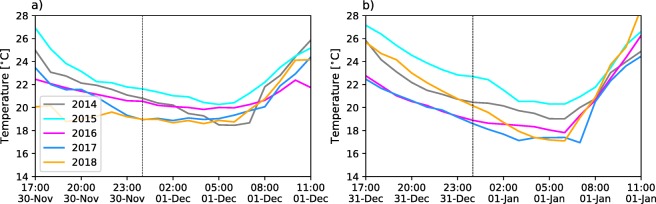
(a, b) Average near-surface air temperature for the bottom of the valley for La Alborada and NYE, respectively. Temperature records from four weather stations located at the bottom of the valley are used to estimate the average

The two cases presented for La Alborada, during 2015 and 2018, exhibit marked differences in the inversion magnitude. During 2015, the inversion layer below 1000 m is weak and starts around November 30, 18:00 LT and ends December 1, at approximately 08:00 LT. In this case, the aerosol load resulting from the fireworks is quickly dispersed after 08:00 LT and the structure of the layer during the night is not as homogeneous and stationary as that in other cases. This is evident in Fig. 14b, where the BI considerably decreases after 08:00 LT, and the vertical structure during the night is highly unstable, with a variable height of the top of the aerosol load near the surface. In contrast, during 2018, the magnitude of the inversion layer is higher and it is neutralized after December 1, 09:30 LT. As a consequence, as shown in Fig. 14e, the aerosol load remains longer in the ABL and disperses quickly after this time. In the latter case, the aerosol load shows a better organized stable layer, with a nearly constant height of the top of the aerosol load. Average near-surface air temperature records from weather stations at the bottom of the valley (Fig. 17a), for the different years, show consistent evidence that the atmosphere was colder near the surface during 2018 compared with during 2015, leading to a steeper inversion layer, thus extending the lifetime of the overnight stable layer as well as the aerosol residence time.

Similar behavior is observed in the NYE cases presented in Fig. 16 for 2015, 2017, and 2018, with the inversion layer modulating the dispersion and recession of the aerosol concentration curves. The extent of the lower-troposphere stability is considerably greater during 2017 and 2018 than during 2015, with 2017 exhibiting the higher vertical gradient of potential temperature. Again, the average near-surface air temperature records (Fig. 17b), for the different years, show consistently, that the atmosphere was colder near the surface during 2017 compared with during 2015 and 2018, with 2015 showing the warmest conditions among the 3 years, lead to a larger magnitude of the stability during 2017 NYE. BI profiles in Fig. 15d and e, for 2017 and 2018, respectively, also show a distinctly stratified structure in the lower atmosphere, with the aerosol load confined to the lower 500 to 600 m, while in Fig. 15 b, for 2015, shows vertically penetrating aerosol plumes up to 1000–1500 m above the surface, allowing for the dispersion of most aerosols before January 1, 8:30 LT. As previously mentioned, Fig. 7 b shows a slower aerosol load recession during 2017 than that during 2018. This is because of the larger and extended potential temperature gradient during 2017 as shown in Fig. 16d.

### Near-surface and vertical profiles of winds

**Fig. 18:**
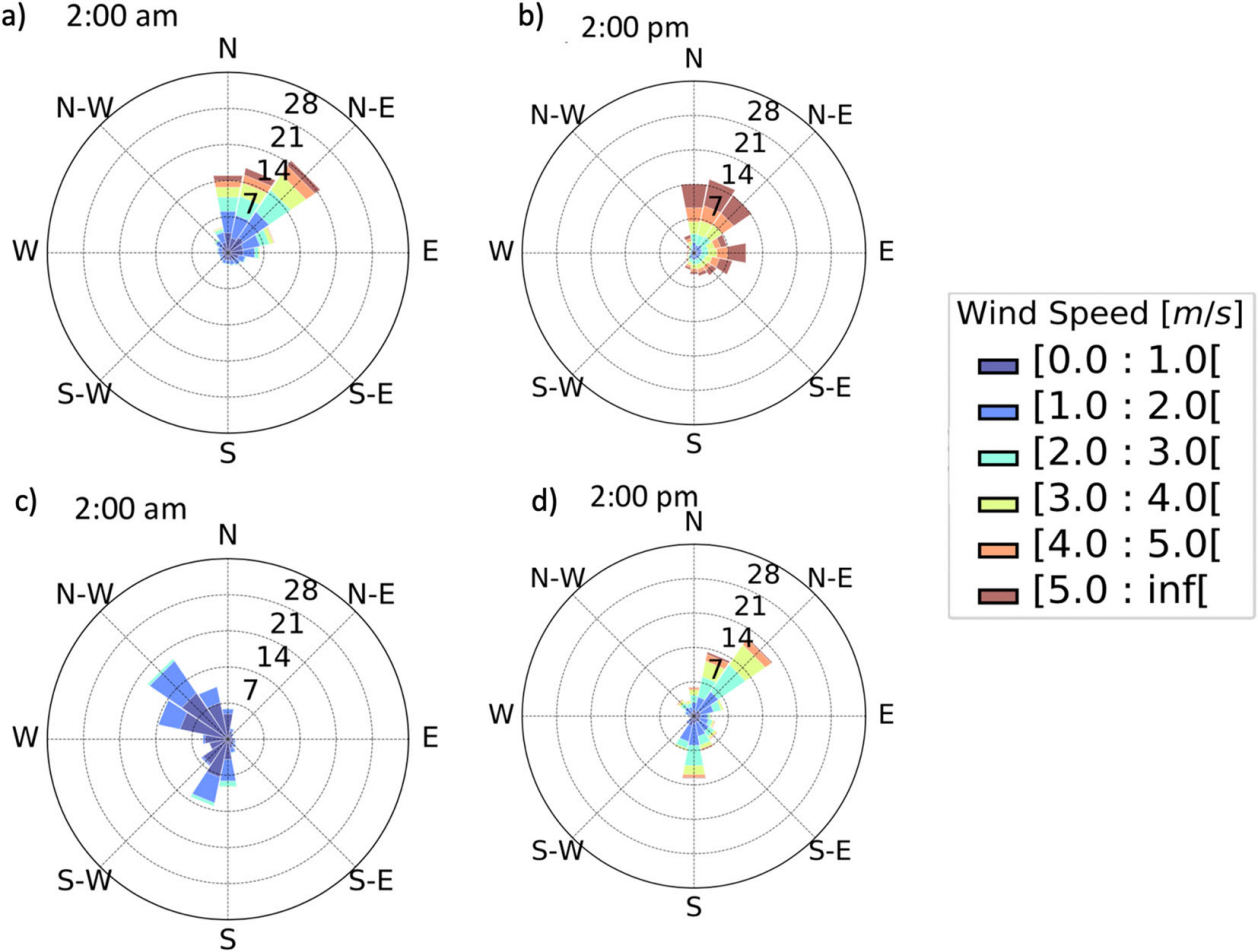
(a) and (b) Long-term wind roses at SIATA main operations building located at the base of the valley (MWR site), for the period from (a) 02:00 to 03:00 LT, and for the period from (b) 14:00 to 15:00 LT. (c, d) Similar to (a) and (b) for a weather station located over the western hill

As we argue in the previous subsections, vertical dispersion and rainfall-triggered aerosol removal are especially important in narrow valleys such as the Aburrá Valley, where the complex topography limits the horizontal advection of pollution due to the magnitude of surface winds, which is usually very weak. Atmospheric stability conditions, the evolution of the atmospheric boundary layer, and, in particular, the development of a deep convective layer are considered determining factors in pollutant concentrations in the Aburrá Valley (Herrera-Mejía and Hoyos 2019). A stable atmosphere inhibits atmospheric vertical exchanges and favors pollutant accumulation, while unstable environments promote pollutant dispersion and mixing (Whiteman 2000). Figure 18 shows the long-term wind roses at SIATA main operation building located at the base of the valley and a location over the western hill, for the period from 02:00 to 03:00 LT, and from 14:00 to 15:00 LT. In general, the roses for the 02:00–03:00 period, key for dispersing fireworks-related pollutants show very weak winds, with magnitudes typically less than 2 ms^−1^. During the afternoon, the magnitude of the winds increases; however, it is not useful for dispersing aerosols due to timing. On the base of the valley, the winds are from the northeast, day and night, following the preferential alignment of the valley. Over the western hill, winds blow downhill from the the northwest during nighttime and uphill from the northeast during daytime. The preferential wind direction for the valley hills during nighttime, which tends to be towards the center of the valley, precludes fireworks-related aerosol export uphill. Over the eastern hill (not shown), the wind direction is reversed, and the magnitude is very similar. During La Alborada and NYE, from 2014 to 2018, the wind speed at the SIATA site (bottom of the valley) is less than 2.5 ms^−1^. Near-surface evidence suggests that, around midnight, the wind speed is weaker for years with stronger inversion layers (larger positive vertical gradients of potential temperature), which exacerbates the fireworks-related pollution accumulation during a more stable atmosphere. For example, during La Alborada 2015 and during NYE 2017 and 2018, the wind speed is less than 1.5 ms^−1^.

**Fig. 19:**
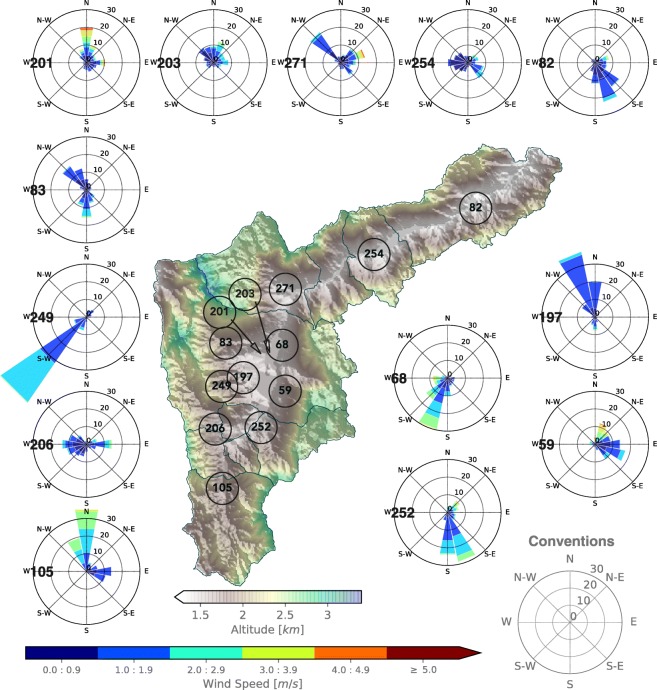
Wind roses for all available stations along and across the valley from 23:00 LT December 21, 2018 to 01:00LT January 1, 2019

**Fig. 20:**
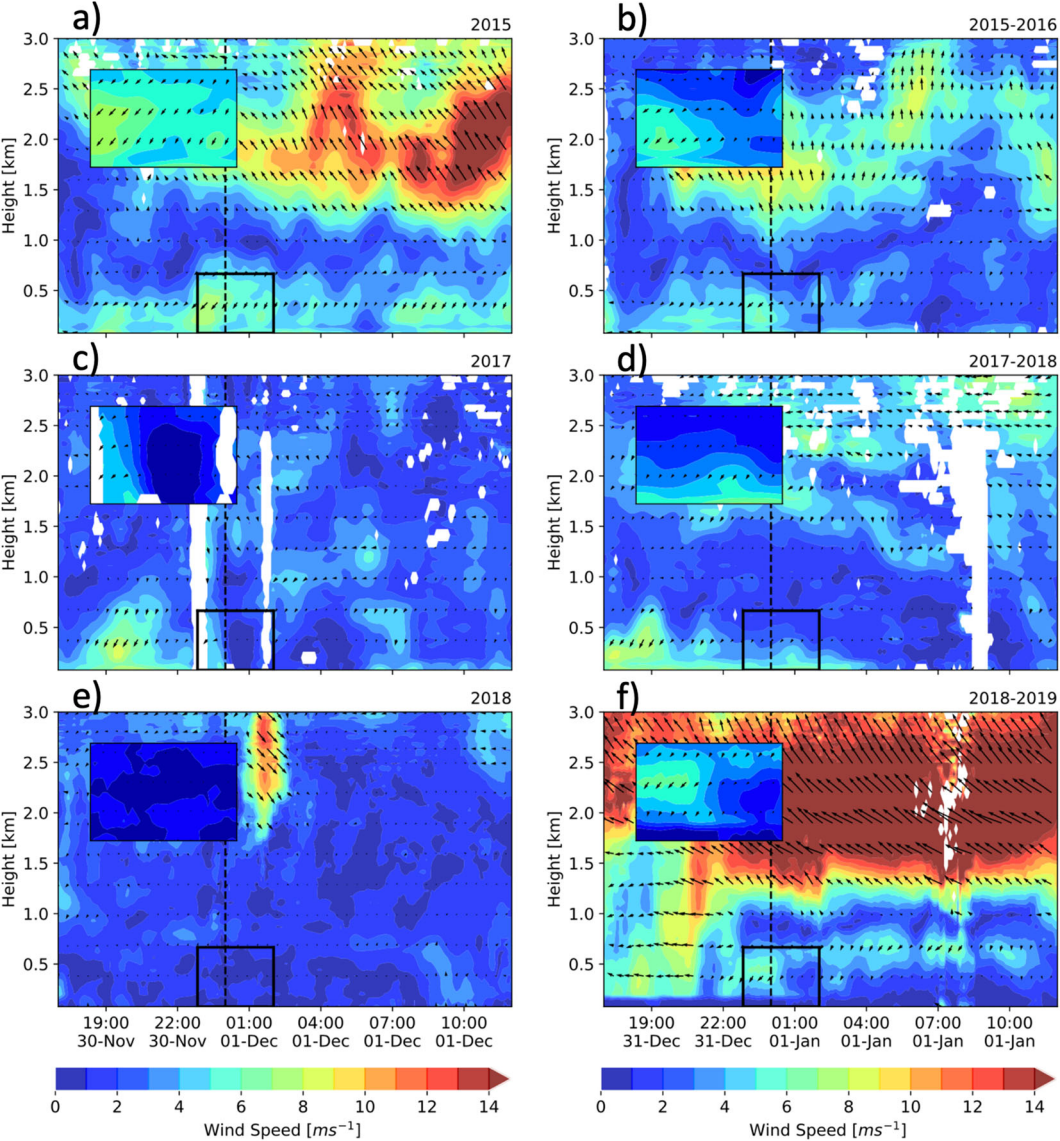
(a) and (b) Lower troposphere (0–3000 m above the surface) wind profiles for La Alborada and NYE cases during 2015, respectively. Wind profiles are retrieved using the RWP. The time domain spans from November 30, 17:00 LT, to December 1, 12:00 LT, and December 31, 17:00 LT, to January 1, 12:00 LT, to highlight the wind profile associated with the changes in aerosol concentration associated with fireworks. The colors represent wind speeds and the arrows the horizontal wind direction. The insert shows a zoom of the wind profiles around 00:00 LT, for the first 500 m above the surface. c–f Similar to (a) and (b) for 2017, and 2018, respectively

The low wind speeds at night, within the valley, are not evident in just a few selected stations, but rather in all of them. Figure 19 shows the wind roses for all available stations along and across the valley from 23:00 LT December 21, 2018, to 01:00LT January 1, 2019. It is clear that, most of the time, the magnitude of the wind is less than 2.0 ms^−1^ in all the stations. Winds during La Alborada 2018 are even calmer than those shown in Fig. 19. Calm wind speeds at night are also present in the entire vertical extension of the ABL and also below the valley divide. Figure 20 presents the lower troposphere wind profiles for La Alborada and NYE cases during 2015, 2017, and 2018, showing evidence of the weak winds in the ABL. From the examples in the figure, 2015 corresponds to the largest magnitude of near-surface wind speeds, which is consistent with the stability assessment. Wind speeds during 2015 NYE are of a smaller magnitude than those during La Alborada, as a result of a more stable environments. La Alborada and NYE cases for 2017 and 2018 show significantly weaker winds, also consistently with the profiles of potential temperature (Fig. 14). The magnitudes shown in Figure 20 does not promote an efficient aerosol removal from the valley. The lower-troposphere northeasterlies during the 2018 NYE (Fig. 20f) might explain the fact that PM2.5 anomalies increase from east to west.

## Conclusions

In Medellín and its metropolitan area, the use of fireworks is widespread during the Christmas season, and in particular, during La Alborada and NYE, causing a deterioration in ambient air quality. The effects of La Alborada and NYE fireworks on PM concentration in the Aburrá Valley were assessed using records from the official air-quality monitoring network and a low-cost PM citizen science network, BI retrievals from a ceilometer network, a MWR to characterize the atmospheric stability, and a RWP to evaluate the dynamic structure of the atmosphere. We found significant increases in PM2.5 and PM10 mass concentrations resulting from the use of fireworks associated with the La Alborada and NYE celebrations.

The hourly evolution of the fine and coarse PM for the 2015 to 2018 period, during the La Alborada events, shows PM changes ranging from large concentration increments to almost no perceptible air-quality effects, depending on the location of the monitoring station and the meteorological conditions. Densely populated communes, with relatively low quality-of-life conditions, show hourly PM2.5 concentration increments ranging from 50 to 100 μgm^−3^. Areas with low population density do not show important PM increments after the La Alborada midnight. Overall, the range of the La Alborada PM2.5 increments is from as low as 7 to as high as 100 μgm^−3^, with significant variability from year to year depending on meteorological conditions and social factors. Precarious outdoor conditions due to weather events discourage people in the region from using fireworks. During NYE celebrations, the change in concentrations around midnight is more notable than that during La Alborada. NYE hourly anomalies are as high as 150 μgm^−3^ when compared with the previous day and up to 190 μgm^−3^ when compared with the concentration levels at approximately 18:00 LT, on December 31.

The high density of the low-cost PM citizen science monitoring network allows one to unequivocally identify the communes with the highest PM increments resulting from fireworks. The spatial distribution of the PM2.5 concentration anomalies during La Alborada shows that the largest PM changes are confined to Medellín, in particular to the eastern hill communes, with the exception of the highest quality of life area. The northeastern Medellín communes show an average increase greater than 42 μgm^−3^, while the western communes show an average increase of nearly 27 μgm^−3^. The increase during NYE is greater than that during La Alborada, and it is observed not only in Medellín but also in all the municipalities in the metropolitan area. The median increments relative to the 22:00 LT concentrations, which correspond to a representative value for the entire valley, are 15 and 20 μgm^−3^ during La Alborada and NYE, respectively. Despite the cited educational campaigns and bans to reduce fireworks use, the year with the largest anomalies in PM2.5 concentration since 2015 is 2018. These anomalies are larger than the increments resulting from fireworks reported in most cities throughout the world.

The observed PM10 and PM2.5 concentration changes during La Alborada and NYE suggest that most of the PM mass increments associated with fireworks correspond to fine particles. The PM increments resulting from fireworks show a different behavior compared to rush hour PM peaks on a regular weekday, with almost no increase in the net amount of BC in the atmosphere. Another difference between fireworks-induced PM increments and those resulting from direct combustion engine vehicle emissions is the mass percentage of ultrafine particles; in addition to higher BC content, fossil fuel–powered vehicles generate a higher content of ultrafine particles compared to that of fireworks.

The vertical structure of the aerosol plumes from fireworks during La Alborada and NYE, and their residence time in the atmosphere, strongly depends on the structure of the ABL, which in turn is modulated by the vertical gradient of the potential temperature. In other words, the prolongation of the nocturnal stable layer determines the aerosol load recession. A pronounced thermal inversion leads to a slower dispersion and recession of the aerosol concentration. Ceilometer BI profiles show a substantial change immediately after the La Alborada and NYE midnights, confined to the first 550 m, which is precisely the height of the ABL at the base of the Aburrá Valley, and acts as a lid limiting the vertical dispersion of the pollutants. Under stagnant conditions, and given the topographic setting of Medellín, aerosols emitted during the use of fireworks are sufficient to significantly increase the PM concentration in the entire ABL. Under strong inversion conditions, aerosols present evidence of a marked vertical gradient, with the increments in BI being fairly homogeneous within the ABL, lasting until the onset of the CBL. Weak thermal inversions lead to a fast dispersion of aerosols, with aerosol plumes from fireworks episodically penetrating above the ABL.

## Footnotes

1 See reports by CNN and BBC as examples (accessed September 5, 2019):

- https://edition.cnn.com/2016/11/29/football/brazil-chapecoense-plane-crash/index.html
- https://www.bbc.com/news/world-latin-america-38155840

2 See examples reported in (accessed September 5, 2019):

- https://www.elespectador.com/noticias/nacional/antioquia/no-haya-alborada-peticion-del-alcalde-de-medellin-articulo-668215
- https://www.elcolombiano.com/antioquia/piden-cancelar-alborada-en-solidaridad-a-victimas-de-chapecoense-FA5471236

3 See reports by (accessed September 5, 2019):

- https://www.elcolombiano.com/antioquia/el-llamado-es-a-no-quemar-polvora-durante-la-alborada-CH7786994
- https://www.publimetro.co/co/medellin/2016/12/01/primera-vez-alborada-no-anos-anteriores-medellin.html
- https://www.eltiempo.com/colombia/medellin/no-hubo-quemados-durante-alborada-28739
- https://noticias.caracoltv.com/medellin/primera-vez-en-cinco-anos-que-la-alborada-no-deja-personas-quemadas-en-medellin
- https://telemedellin.tv/cero-quemados-alborada/154307/
- http://www.colombiainforma.info/enpiedepagina/2018/11/30/medellin-polvora-y-su-candente-alborada/
- https://www.vivirenelpoblado.com/que-viva-la-arbolada/

4 See for example: https://www.metropol.gov.co/planeacion/Paginas/salud-publica/Huellaton.aspx
